# Conversion of Contaminated Post-Consumer Polyethylene Terephthalate into a Thermoset Alkyd Coating Using Biosourced Monomers

**DOI:** 10.1021/acssuschemeng.3c07560

**Published:** 2024-04-18

**Authors:** Bradley Thomas, Nicole D. A. Lopez, James Railton, Jamal Bousbaa, Justin J. B. Perry, Matthew G. Unthank

**Affiliations:** Department of Applied Science, Northumbria University, Newcastle upon Tyne NE1 8ST, U.K.

**Keywords:** coatings, sustainability, sustainable materials, plastic waste, PET waste, recycling, alkyds, alkyd resin, unsaturated polyester, thermoset, polymers, network polymers

## Abstract

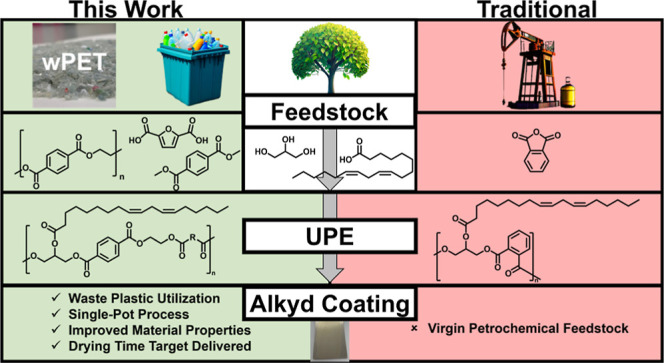

The synthesis of a high-performance oxidative cross-linked thermoset alkyd coating is described that utilizes a novel recycling strategy from contaminated postconsumer waste polyethylene terephthalate (wPET). A single-stage “depolymerization-repolymerization” process has been developed that allows the exploitation of a waste stream from a commercial PET recycling process with 95% efficiency, which, when copolymerized with glycerol and tall oil fatty acid, delivers a sustainable fatty acid-functional polyester suitable for use in thermoset alkyd coatings. Physical drying challenges have been tackled via the development of a convergent polymer formulation strategy from a single source of wPET and the formulation of the resulting fatty acid-functional polymers with commercial alkyd driers, delivering a thermoset alkyd coating suitable for industrial applications.

## Abstract 2

Conversion of Contaminated Post-Consumer PET into a Thermoset Alkyd Coating Using Biosourced Monomers

## Introduction

Self-drying or “oxidative-drying” polyesters based on unsaturated fatty acids have a long and established track-record for use in industrial alkyd coatings.^1^ Thermoset polymers and coatings are those, which undergo a permanent, typically nonreversible covalent network formation during the cure or drying process, delivering a range of excellent properties including mechanical strength, hardness, chemical and solvent resistance,^2^ water repellence/hydrophobicity, and other desirable coating properties.^3^ Alkyd thermoset coatings are used on a large industrial scale, with global consumption reported as high as 1 million tons per annum.^4^

The unique benefit of alkyd thermoset coatings is that they are based on the oxidative-drying of fatty acid-functional polyesters, which are a subclass of unsaturated polyesters (UPEs), where the “unsaturation” is located on the pendant fatty acid chains. These are single-component coating systems, benefiting from simple application processes, long application windows, and long shelf-lives, yet are still capable of delivering the performance properties of a thermoset coating (which are typically two component systems, with limited application windows and preapplication mixing requirements). Such polyesters synthesized from unsaturated fatty acids (e.g., tall oil), a polyol (typically pentaerythritol or glycerol), and phthalic anhydride form the major binder component of commercial alkyd coatings, typically representing 30–50% of a formulated coating.^5^ These coatings are manufactured on a huge commercial scale, and improving sustainability through the minimization or recycling of resources would be impactful across a wide range of industries. Of the monomers used in the manufacture of polymers for alkyd coatings, both glycerol and (tall oil) fatty acids are biosourced. The glycerol is used as a branching agent in the polyester backbone, increasing the average functionality of the polyester and assisting in the chemical drying (cross-linking) process. The fatty acid component delivers the required unsaturated functionality for oxidative cross-linking in the form of “double allylic” groups along the aliphatic chain. In a glycerol branched system, phthalic anhydride is the only major petrochemical-derived monomer used in the polymer manufacturing process, typically constituting between 20 and 30% of the total polymer mass.^6^ Phthalic anhydride is included to promote physical drying by increasing the hardness, glass transition temperature (*T*_g_), and hydrophobicity of the resulting coating and therefore improves the essential coating properties required for a high performance industrial coating (including abrasion resistance, durability, and hardness). Polyethylene terephthalate (PET) is the most widely recycled commodity plastic and contains terephthalic acid as the major constituent by weight, which is a similar (isomeric) aromatic diacid to phthalic acid, the hydrated form of phthalic anhydride. In principle, this molecular building block could be used as a replacement in the manufacture of polymers (UPEs) for industrial alkyd coatings.

Closed-loop recycling processes are well established for PET and the use of recycled PET (rPET) directly is unlikely to result in an improvement in overall sustainability credentials.^7^ However, it has been recently identified (via UKRI Transforming the Foundation Industries Hub, TransFIRe) that during commercial-scale PET recycling, large quantities of small particle size (0.5–3 mm)-contaminated PET plastic flake are produced during the separation and washing steps. This waste PET material (wPET) contains 90–99% PET flake with 1–10% polyolefin contamination. These so-called “flake and wash losses” are estimated to be 157 k tonnes of PET per annum lost from the closed-loop recycling process in the EU alone.^8^ This grade of wPET cannot be further refined or recycled due to its degree of contamination and small particle size, and it is currently sent to landfills or incinerated for energy recovery.

Within the scientific literature,^9−12^ several examples exist of alkyd-type polyesters derived from PET, however, these processes use rPET (not wPET) and typically follow the same pattern of separate steps: (i) conversion of rPET to hydroxyl functional monomers via glycolysis, (ii) separation/purification of monomers, and (iii) a polymerization process still utilizing petrochemical-derived diacids such as phthalic anhydride. Indeed, wPET is an ideal material for the synthesis of such alkyd-type polymers with improved sustainability credentials. Therefore, we propose that if a “depolymerization-repolymerization” process could be developed with wPET, glycerol, and tall oil fatty acid, then this would represent the first example of an alkyd-type “unsaturated” polyester suitable for use in oxidative drying alkyd coating systems, based entirely on biosourced monomers and nonrecyclable contaminated postconsumer wPET, in a one-pot system without the need for additional purification steps.

## Experimental Section

### Materials

wPET flake material contaminated with <2 wt % polyolefins was provided from a mechanical recycling process by Jayplas (Corby, UK), all other materials were purchased from commercial vendors or supplied by industrial partners, as listed in the Supporting Information and used as received. All solvents used were standard laboratory grade and supplied by Fisher Scientific.

### Preparation of Conventional Fatty Acid Functional “Unsaturated” Polyester (UPE-C)

Eleven g of glycerol, 34 g of tall oil fatty acids, and 13.5 g of phthalic anhydride were charged to a 250 mL round-bottom flask, and a Dean–Stark trap was fitted to the flask with a Liebig condenser and nitrogen bubbler. The flask was wrapped in glass wool and aluminum foil, and the reaction was heated at 180 °C for 3 h with 300 rpm stirring before cooling to room temperature. The acid value of the resulting polymer was determined as 16.6 mg of KOH/g in accordance with ASTM D974.

### Preparation of wPET-Derived Fatty Acid-Functional “Unsaturated” Polyesters (UPE 1-4)

8.73 g of glycerol, 30 g of tall oil fatty acids, 20 g of wPET and additional diacid (0.108 mol equiv –COOH), 50 mL of methanol, and 0.5 wt % zinc acetate were charged to a 250 mL round-bottom flask. A Liebig condenser and nitrogen bubbler were connected to the flask, and the mixture was refluxed at 150 °C for two h with 300 rpm stirring, before cooling to room temperature. A Dean–Stark trap was fitted between the flask and condenser, the flask was wrapped in glass wool and aluminum foil, and the temperature was increased to 180 °C. Once the excess methanol had been removed by distillation, the temperature was increased to 250 °C with 180 rpm stirring and left to reflux for 16 h. The reaction was then cooled to room temperature, and any material on the sides of the flask was washed into the bulk with a small volume of toluene. A Claisen adaptor was fitted to the flask with a glass stopper, and the condenser was setup for vacuum distillation with a 100 mL receiving flask. ∼80 mbar vacuum was activated, and the temperature increased to 80 °C, until no toluene was visibly entering the receiving flask. The temperature was then increased to 160 °C and left for 1 h to remove any residual volatiles. The polymer was then cooled, dissolved in toluene, and filtered through Celite to remove polyolefin contamination originally present in wPET, before the toluene was reclaimed using a rotary evaporator. The resulting materials (UPE1-4) were all homogeneous viscous liquids.

### Preparation of Dimethylterephthalate from wPET

20 g portion of wPET, 30 mL of methanol, and 0.2 g of zinc acetate were added to a 60 mL glass pressure vial with a 10 × 6 mm elliptical stir bar. The vial was placed into an oil bath at 120 °C with 300 rpm stirring and left to react for 2 h. The reaction mixture was cooled before pouring into 200 mL of 5 °C deionized water, at which point a crystalline white precipitate formed. The precipitate was filtered and recrystallized in a minimum amount of boiling deionized water to give dimethylterephthalate as the final product.

### Alkyd Coating Formulation (ALK-C and ALK 1-4)

The fatty acid-functional UPEs (UPE-C and UPE1-4) were diluted with white spirit and combined with the drying catalysts and antiskinning agents according to Table S3 in the Supporting Information. This mixture represents the alkyd coating formulations ALK-C and ALK 1-4, respectively.

### Preparation of Coated Panels for Hardness Testing

Two sets of coated panels were prepared for each alkyd-coating formulation; one set was prepared where the coated panels were heated overnight at 120 °C to facilitate drying, and a second set was prepared at room temperature using methyl ethyl ketone peroxide (MEKP, 5 wt %) to accelerate cure. Both sets of samples were applied at a wet film thickness of 100 μm on an aluminum Q-panel (a standardized substrate for assessment of paint and coating systems) and stored at ambient conditions for 1 week prior to testing.

### Vickers Microhardness

Indentations were made using a Buehler MHT-1B microhardness tester with a diamond indenter at 50 g load for a period of 8 s. Indentation areas were measured on an Alicona Infinite Focus G5 digital microscope.

### Preparation of Tensile Test Specimens

Alkyd-free films were prepared according to the formulations in Table S3 in the Supporting Information, with the exclusion of white spirit, and were drawn down on to an aluminum panel coated with PTFE film to a wet film dimension of 150 × 300 × 0.4 mm before being cured at 120 °C overnight. The films were released from the sheet using a thin microspatula to provide free-films of cured polymeric coatings. Samples were cut to size using a laser cutter to ISO 1BA tensile specimen dimensions by using a Glowforge Basic laser cutter equipped with a 40 W CO_2_ laser. Test specimen thicknesses were measured using a digital micrometer.

### Tensile Testing

Tensile tests were performed on an Instron 5969 universal testing machine at a grip separation speed of 10 mm/min, and 50 point moving averages were applied to the load data before calculating stress. Young’s modulus was determined as the slope of the stress/strain curve between 0.5 and 1% strain.

### Gel Permeation Chromatography

Polymers were dissolved in tetrahydrofuran (THF) at a concentration of 2 mg/mL and filtered through 0.2 μm nylon syringe filters. Samples were analyzed using an Agilent 1260 infinity II system equipped with a refractive index and viscometry dual detection suite, fitted with 1 × 50 mm PLgel MiniMIX-D 5 μm guard column, 1 × 250 mm PLgel MiniMIX-D 5 μm column, and 1 × 250 mm PLgel MiniMIX-E 3 μm columns in sequence at 50 °C, using a THF mobile phase and a flow rate of 0.5 mL/min. Molecular weight analysis was performed against a calibration curve of polystyrene standards (EasiVial PS-M and PS-L supplied by Agilent).

### Attenuated Total Reflectance-Fourier Transform Infrared Spectroscopy

Infrared spectroscopy was performed on a Bruker Alpha Platinum-attenuated total reflectance (ATR) instrument, and the output data were analyzed in OPUS software. Transmittance minima were expressed in wavenumbers (cm^–1^).

### Contact Angle

Contact angle measurements were performed on an Ossila contact angle goniometer using 4 μL of deionized water droplets dispensed from a 50 μL glass micropipet at 20 °C. Images were analyzed using Ossila contact angle software 4.1.0.

### Dry Time Recording

Alkyd coatings were drawn down onto 300 × 25 mm glass plates to a 100 μm wet deposition thickness and assessed on a TQC Sheen dry time recorder with a recording time of 72 h at 20 °C. Drying events were determined in accordance with ASTM D5895.

### Nuclear Magnetic Resonance Spectroscopy

Proton nuclear magnetic resonance (NMR) analyses were performed at 25 °C using a JEOL ECS400 Delta spectrometer at a frequency of 399.78 MHz in deuterated chloroform (CDCl_3_). All chemical shifts were quoted as parts per million (ppm) relative to tetramethylsilane (TMS, δ = 0 ppm).

### Thermogravimetric Analysis

Thermogravimetric analysis (TGA) was performed on a PerkinElmer TGA 8000, with a temperature scan from 30 to 600 °C at a heating rate of 20 °C/min.

## Results and Discussion

**Figure 1:**
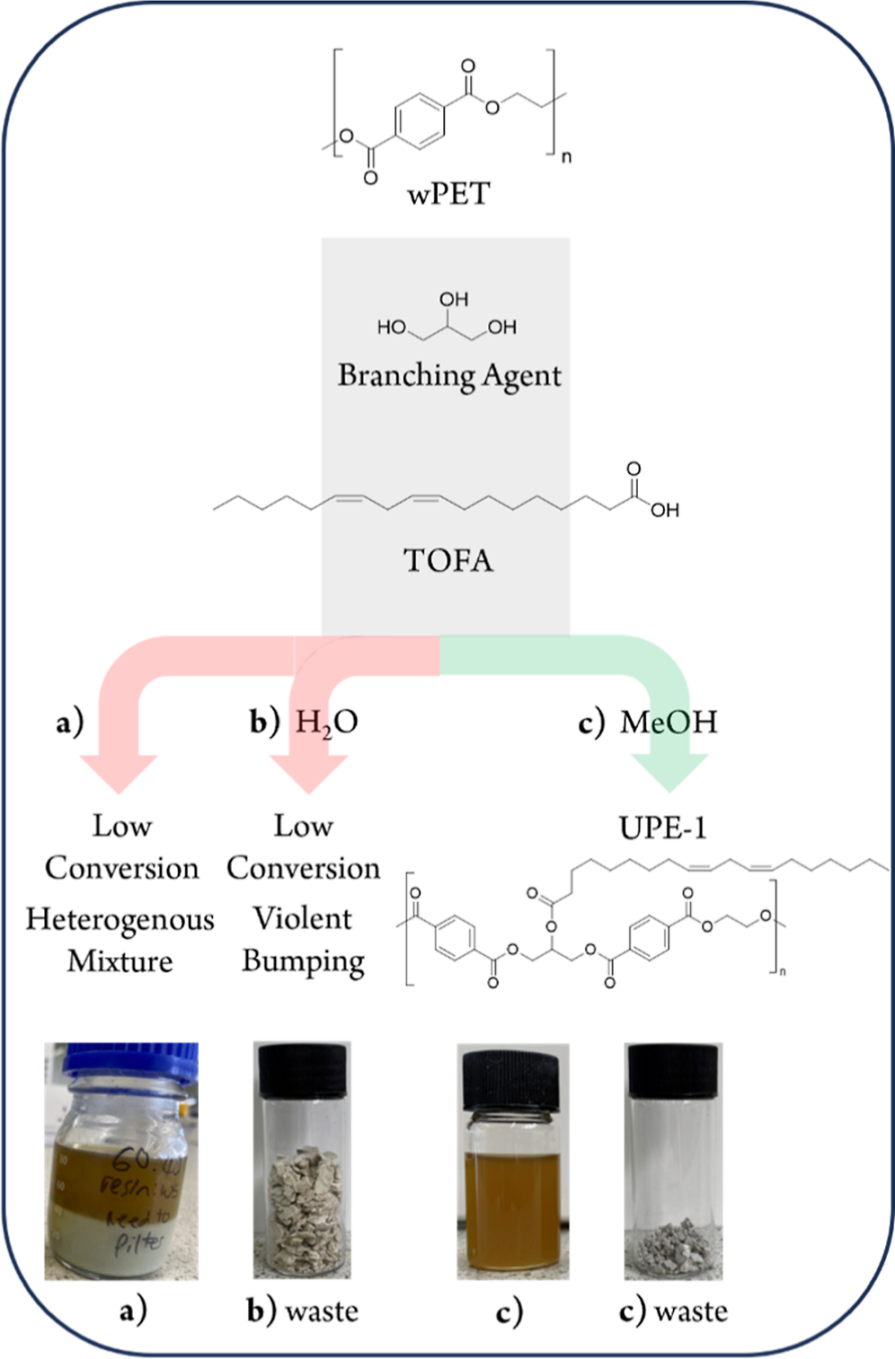
Schematic overview of the process optimization for the synthesis of fatty acid-functional “unsaturated” polyesters (UPE) from wPET. Routes; (a) no solvolysis, (b) hydrolysis, and (c) methanolysis.

Converting wPET into fatty acid-functional UPE’s with low impact processing is the primary objective of this research. Using wPET, tall oil fatty acid, and glycerol as the sole reagents in a depolymerization-repolymerization process would be the most direct synthetic route to this class of polyester. However, despite extensive optimization attempts, the resulting reaction mixture displayed significant heterogeneity, containing substantial quantities of unreacted wPET solid particulate. These processing issues were attributed to the poor solubility of wPET in the tall oil/glycerol reaction mixture (Figure 1, route a). It was therefore hypothesized that in situ solvolysis of wPET into lower molecular weight polymer fragments (oligomers) may assist in the dissolution of wPET in the tall oil/glycerol reaction mixture. This was attempted using water as the solvent (due to its low environmental impact); however, the high melting point of PET^13^ and its relative hydrophobic nature required very high reaction temperatures, resulting in significant processing issues such as violent bumping, discoloration, and ultimately poor conversions of wPET (Figure 1, route b, > 40% mass recovery of wPET). Methanol can be produced from sustainable carbon-containing feedstock, including biogas, biomass, waste streams, and CO_2_^14^ and also has an established role in the chemical depolymerization of PET (i.e., to monomeric dimethyl terephthalate).^15,16^ Such processes have reported advantages over the strong acidic/alkali conditions and high temperatures/pressures that are required for water-mediated solvolysis (i.e., hydrolysis).^15,17^ It was hypothesized that the introduction of methanol to the reaction mixture could facilitate in situ (partial) depolymerization of wPET to more soluble wPET-oligomers, which would improve miscibility in the tall oil/glycerol reaction mixture. This would then allow solution-based repolymerization of the wPET-oligomer with tall oil and glycerol, which could be driven to form high molecular weight polymers through subsequent removal of methanol (and water) via distillation with solvent recovery.

A process was developed by which a wPET/tall oil/glycerol reaction mixture was refluxed in methanol at 150 °C for 2 h before initiating distillation to remove the methanol. These conditions were selected in order to exceed the *T*_g_ of PET and increase the rate of formation of methyl esters during methanolysis of the PET. The resulting one-pot process delivered a significant improvement in wPET repolymerization efficiency, increasing the conversion of wPET from <60% (aqueous process, Figure 1, route b) to >95% (methanolic process), as illustrated in Figure 1, route c. The resulting polyester isolated from the methanol-mediated repolymerization process delivered a wPET/tall oil fatty acids/glycerol-based polymer (UPE-1), which is derived completely from waste and biobased feedstocks, allowing for a highly resource-efficient functional polymer for coating applications.

**Figure 2:**
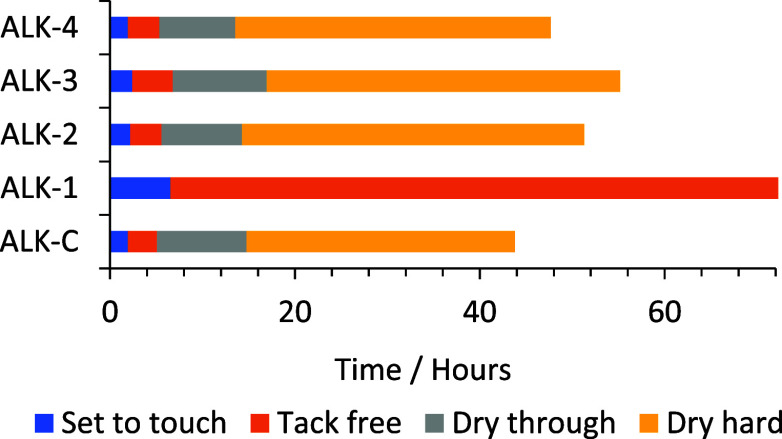
Dry time recording results of alkyd coatings (ALK), including key drying time events across a 72 h testing period.

To evaluate the film forming and drying properties of wPET-derived polyester product UPE-1, the polymer was formulated into an alkyd coating using standard oxidative drying catalysts and solvents typical of commercial alkyd coatings (see Supporting Information, Table S4). The resulting formulated alkyd coating was applied to 300 × 25 mm glass plates, and the key drying time events (set-to-touch, tack-free, dry-through, and dry-hard) were studied using a TQC Sheen dry time recorder. The alkyd coating (ALK-1) derived from UPE-1 exhibited significantly extended (slower) drying times when compared with the control petrochemical-derived alkyd coating (ALK-C) prepared for comparative purposes from control polyester (UPE-C). As illustrated in Figure 2, the “set to touch” time (defined as the point at which the curing film has solidified sufficiently that it no longer flows back behind the recording stylus) of ALK-1 is approximately 3 times longer than that of ALK-C. ALK-1 also did not achieve a tack-free time (defined as the point at which the film surface has cured sufficiently that a continuous track in the film ceases and the recording stylus tears the film) during the 72 h test period, indicating poor drying performance.

**Figure 3:**
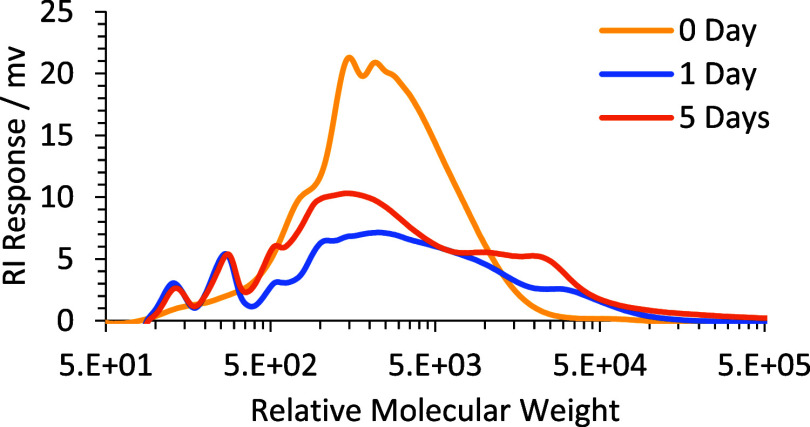
GPC chromatogram of ALK-1 (sol content) after 0, 1, and 5 days drying.

To explore and understand the poor drying performance of ALK-1, the soluble fractions (sol) of the drying coating at days 1 and 5 were extracted with THF, and the molecular weight distributions were characterized by gel permeation chromatography (GPC). Analysis of the sample extracted from the alkyd coating (ALK-1) after both 1 day and 5 days of drying, showed a broadening of the molecular weight distribution by GPC and an increase in average molecular weight, over time (Figure 3). This increase in molecular weight provides evidence of oxidative cure and cross-linking (i.e., the primary chemical drying mechanism of alkyd coatings) but does not explain the lower rate of drying when compared to that of the control (ALK-C).

**Figure 4:**
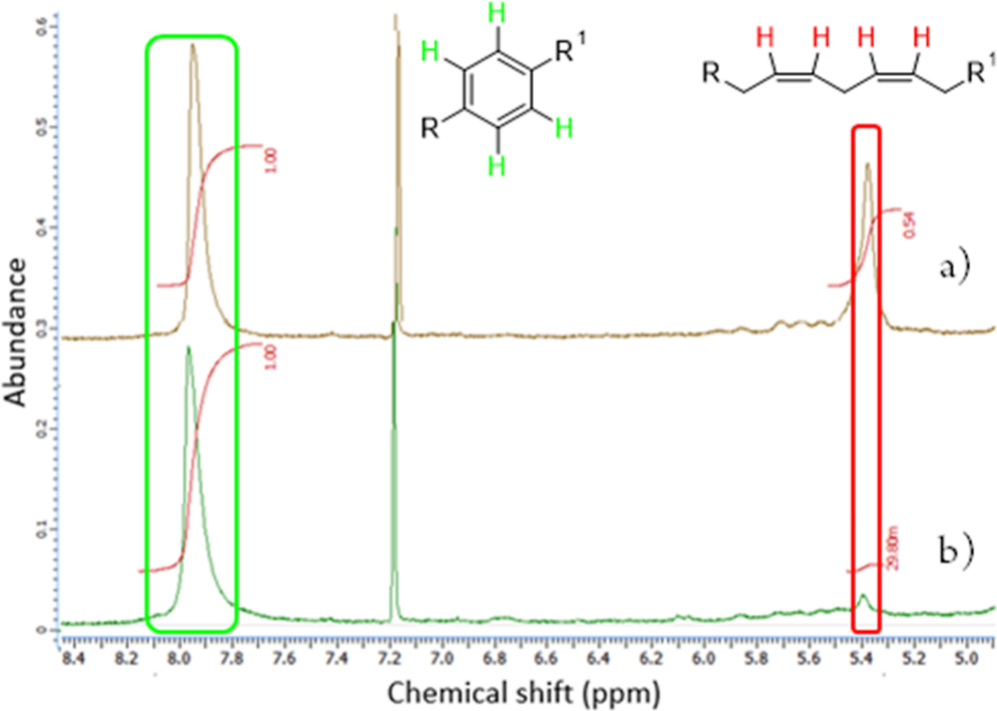
^1^H NMR analysis of ALK-1 (sol content) after (a) 1 and (b) 5 days of drying. Double allylic CH_2_ signal at 5.4 ppm is significantly reduced after 5 days.

The sol-fraction of ALK-1 was also studied by ^1^H NMR (Figure 4) at both days 1 and 5 of the drying process, highlighting significant differences in the chemical composition. The sol-fraction after 1 day showed a high abundance of double allylic CH_2_ protons in the ^1^H NMR, characterized by the broad singlet at 5.3–5.4 ppm^18^ when normalized against the aromatic proton signal at 7.8–8.0 ppm (Figure 4a). The sol-fraction after 5 days in contrast shows a significant reduction (94.5% reduction, Figure 4b) in double allylic CH_2_ proton abundance, signifying the progression of oxidative cross-linking known to occur at these functional group positions in the cure of alkyd-coating systems.^19,20^ Despite this high degree of chemical cross-linking/drying, the coating remained insufficiently hard (tacky) after 3 days of cure (Figure 2, ALK-1). To address this drying performance issue, a method to increase the molecular weight of the wPET-derived polymer product (prior to formulating into an alkyd coating) was required, which would improve the “physical drying” contribution of the polymer to the alkyd coating, improving the resulting material performance, including drying.

It was hypothesized that the poor hardening and drying performance of alkyd coating (ALK-1) was attributed to the low initial molecular weight of UPE-1 (Mw = 3320) when compared with the control polymer UPE-C (Mw = 9680, Table 1, entry 1 vs 2). The molecular weight distribution of step-growth polymerization processes (such as described here), can be modeled using the well-established methodologies of Carothers.^21^ Both conversion of functional groups and relative stoichiometric ratios of reactive functional groups (namely, hydroxyl and carboxylic acid in this case) control the maximum achievable molecular weight distribution. Carothers-based numerical simulation of the control polymer UPE-C provided a theoretical degree of polymerization of 7.48. While it is difficult to determine an accurate value of the extent of reaction (*p*) for UPE-1 (due to the use of methyl ester, not carboxylic acid), if an extent of reaction of 100% was assumed (*p* = 1) and that wPET is considered as contributing equal moles of terephthalic acid (diacid) and 1,2-ethanediol (diol), with a repeat unit molecular weight of 192.2 g/mol, then the theoretical maximum degree of polymerization *X*_n_ for UPE-1 would be 3.96, significantly lower than the calculated value for UPE-C (calculations available on pages S5 and S6 in the Supporting Information). It was concluded that this is due to the monomer formulation used to create UPE-1 having a hydroxyl excess of 63%. This high hydroxyl excess predominantly originates from the contribution of 1,2-ethanediol (diol) from the wPET, with an additional contribution from the glycerol (triol) branching agent. This value is significantly greater than that calculated from the control UPE-C formulation, which showed an 18% hydroxyl excess. The resulting coatings derived from UPE-C had greatly improved hardening and drying performance in comparison to UPE-1-derived coatings (Figure 2, ALK-C vs ALK-1). Since the greater the stoichiometric excess, the more the ultimate achievable molecular weight is limited for any given formulation,^22^ we sought to reduce the hydroxyl excess in the wPET-derived polymer series (UPE-2-4).

**Table 1:** Calculated Average Molecular Weights of the UPEs

entry	sample	Mp	Mn	Mw	PD
1	UPE-C	2370	1620	9680	5.96
2	UPE-1	1190	1310	3320	2.53
3	UPE-2	2610	1600	4910	3.06
4	UPE-3	2280	1450	3990	2.74
5	UPE-4	3540	1980	12,200	6.17

**Figure 5:**
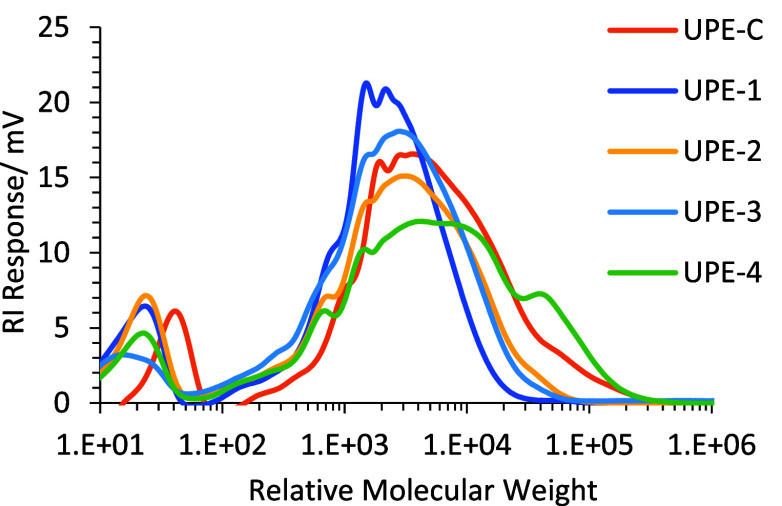
GPC chromatograms of UPE-1 to UPE-4, including control polymer UPE-C.

Phthalic anhydride was therefore tested (as a model reagent only) in the monomer/wPET formulation to reduce the theoretical hydroxyl excess. Due to the nature of the methanolysis process, the polymerization reactions could not be monitored by acid value (as would be typical for polyesterifications).^23^ Instead, polymers were formulated to a theoretical gel point of fractionally over 1 (1.001) to prevent gelation,^21^ and reaction mixtures were processed at temperature for extended periods to ensure maximum possible conversion of functional groups (see Experimental Section for details). The resulting polymer UPE-2 (produced from wPET, glycerol, tall oil fatty acid, and phthalic anhydride) had a theoretical hydroxyl excess of 18%, in line with that of the control (UPE-C). UPE-2 was analyzed by GPC (Figure 5 and Table 1, entry 3) and showed an increased molecular weight distribution when compared with UPE-1, with peak and number-average molecular weights in line with the control UPE-C, and an improved Mw in comparison to UPE-1. UPE-2 was then formulated into an alkyd coating (ALK-2).

Dry time recording analysis of coating ALK-2 (Figure 2, ALK-2) showed significantly improved drying performance (when compared to ALK-1), with key drying event times comparable to those of control UPE-C.

**Figure 6:**
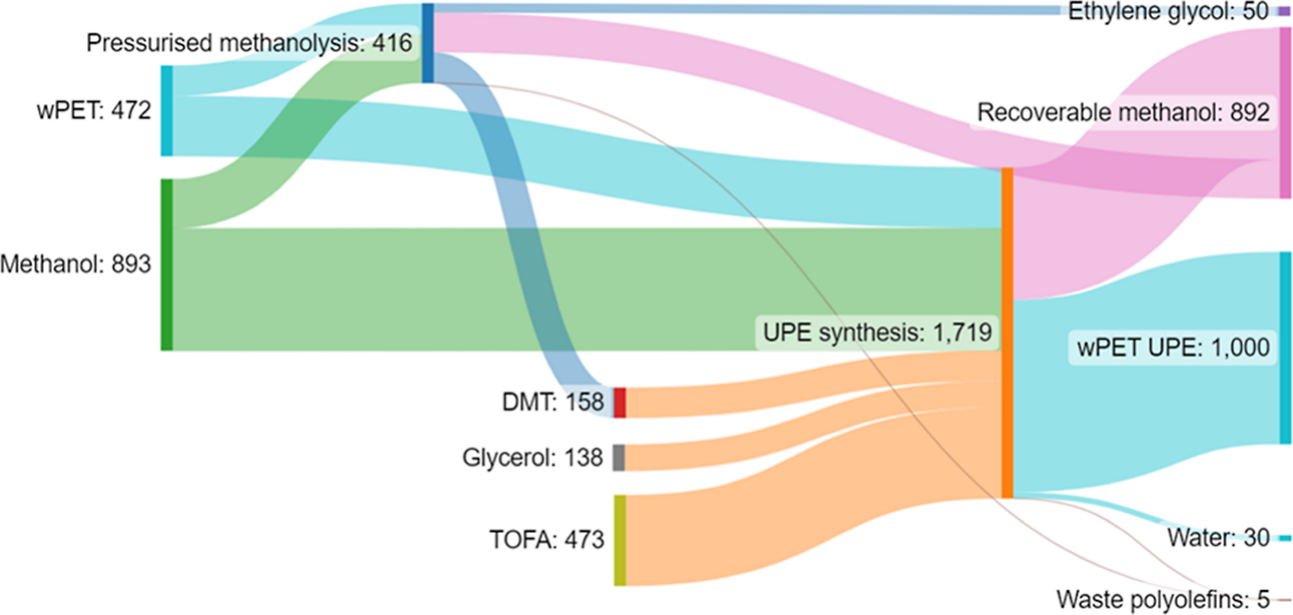
Material flow diagram for the production of 1 kg of wPET-derived UPE-3, utilizing a convergent approach to increase waste-derived content, values expressed in grams, and 100% efficiency assumed.

Despite the measured improvement in drying performance, our aim was that the resulting polymer needed to be 100% based on biosourced or plastic waste feedstocks. Two approaches were identified to maintain the proposed sustainability strategy; (a) to develop a convergent process using wPET as the feedstock, where hydroxyl excess was decreased through the incorporation of a wPET-derived terephthalic ester or (b) to identify and incorporate a suitable biobased diacid available on a large scale at low cost. To explore the first of these approaches (methodology a), the wPET source was used in two complementary depolymerization reactions (one being the pre-existing partial depolymerization and the other being full depolymerization). A process was devised to create a recycled dimethyl terephthalate ester (rDMT) via pressurized methanolysis of wPET. The resulting rDMT was then reintroduced to the original wPET/glycerol/tall oil fatty acid formulation to deliver a polymer with significantly lower hydroxyl excess and a higher molecular weight (UPE-3) with the recovery of methanol. This process is visualized in the material flow diagram in Figure 6, highlighting the masses of reagents required to produce 1 kg of UPE-3, whereby additional monomer is produced from the same contaminated waste stream. In this instance, 158 g of rDMT and 50 g of ethylene glycol would be produced in the pressurized methanolysis step, and the rDMT is then recharged to the main polymer synthesis process.

**Figure 7:**
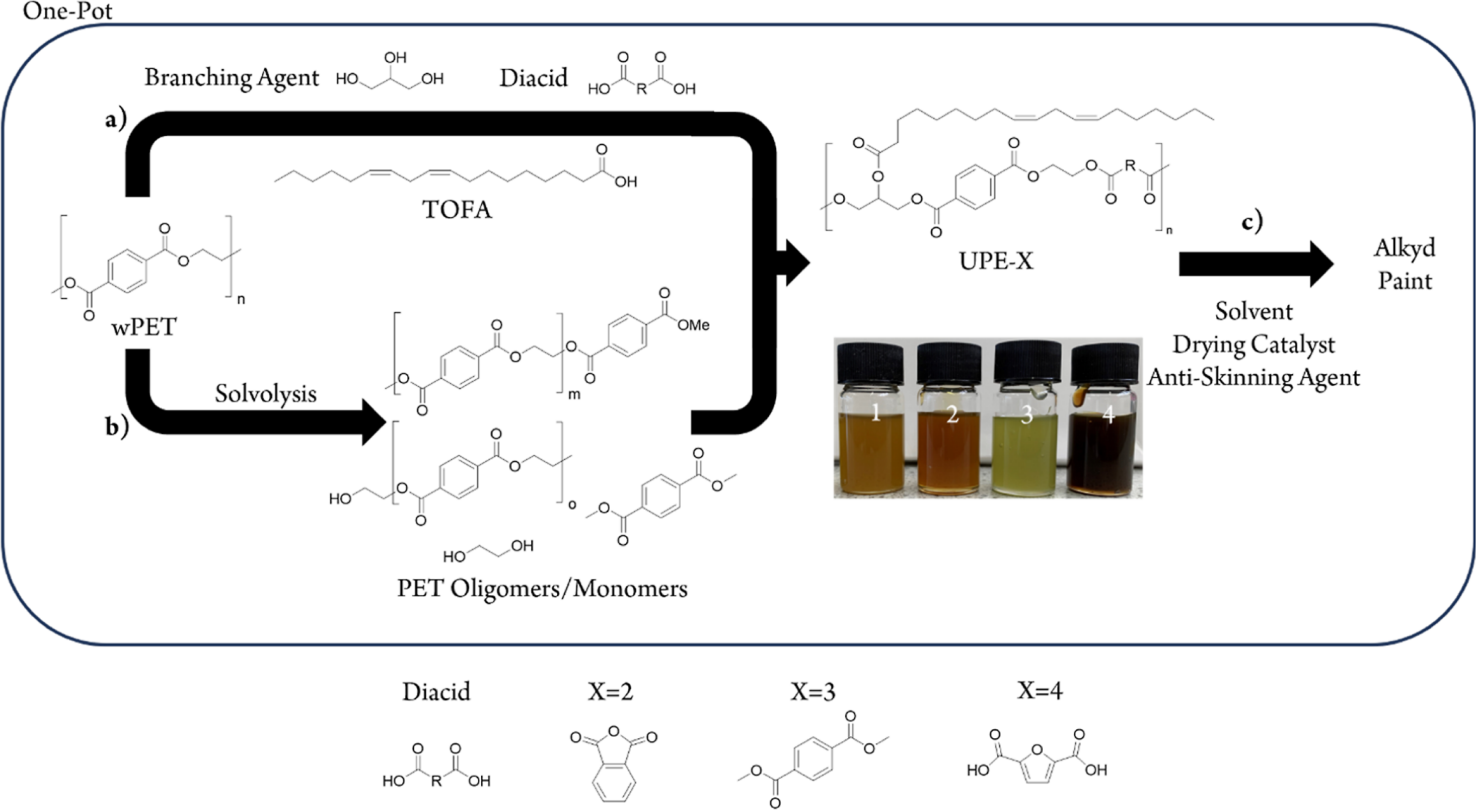
Scheme for the one-pot synthesis of UPEs with additional diacids. Steps (a) and (b) occur simultaneously for wPET conversion to UPEs. *X* = 2, 3, and 4 used diacids phthalic anhydride, DMT, FDCA, respectively. UPE-1 was formulated without additional diacid.

The approach of methodology b, was to use a biobased diacid to reduce hydroxyl excess. 2,5-Furan dicarboxylic acid (FDCA, Figure 7, diacid 4) is widely regarded as an emerging biosourced aromatic diacid,^24,25^ produced from biomass-derived 5-hydroxymethylfurfural.^26,27^ Similar to the use of rDMT, FDCA can be introduced to the main polymerization process with wPET, glycerol, tall oil fatty acid, and methanol, in a calculated proportion to reduce the hydroxyl excess to a value in line with UPE-C. The processing conditions for all reactions were the same as those used in the synthesis of UPE-1 (see Experimental Section). A general scheme is displayed in Figure 7. For comparative purposes, the phthalic anhydride (petrochemical-derived)-modified polymer UPE-2 was also included in the physical property evaluation study.

The resulting fatty acid-functional polyesters (UPE-2 to UPE-4 and UPE-C) were analyzed via GPC (Figure 5) to study the impact of the formulation changes on the resulting polymer molecular weight distribution. The results demonstrated that the polyesters (UPE 2-4) formulated with reduced hydroxyl excess, all measured increased number (Mn) and weight (Mw) average molecular weights in comparison to the original wPET-only-based prototype polyester (UPE-1). UPE-2 and UPE-3 exhibit similar values for molecular weight and polydispersity (Table 1, entry 3 vs entry 4), while UPE-4 (utilizing FDCA) exhibited a significantly higher Mw and PD value (Table 1, entry 5). This higher molecular weight distribution (with FDCA) may be assigned to side reactions (such as ring hydrolysis) of the FDCA facilitated by the high processing temperatures required for copolymerization with wPET. High processing temperatures in polycondensations with FDCA are also known to lead to side reactions and discoloration,^28,29^ which is consistent with the dark discoloration observed in UPE-4 (Figure 7, UPE-4 picture).

The polyesters UPE-C and UPE 1-4 were formulated into alkyd coatings (ALK-C and ALK 1-4) (see Supporting Information for details) and compared directly for drying/hardening times using a TQC Sheen dry time recorder (Figure 2). All the higher molecular weight wPET-based polyesters (UPE 2-4) showed significantly improved drying performance vs the lower molecular weight UPE-1 polyester. This is especially significant for the rDMT and FDCA-based polyesters (UPE-3 and UPE-4, respectively) since these are 100% waste and bioderived. The hard-dry times for UPE-2, -3, and -4 correlate well to the Mp, Mn, and Mw values, with the higher molecular weight distribution polymer UPE-4, achieving hard-dry in the shortest time, followed by UPE-2 then UPE-3.

The improved drying performance of UPEs 2-4 can be attributed to two main effects; (i) the higher initial molecular weight distributions of the polymers, delivering an improved contribution to “physical drying” in the formulated alkyd coatings (ALK-2, -3, and -4, respectively) and (ii) the higher average functionality of the resulting polymers (i.e., higher molecular weight polymers will contain a relatively higher average functionality of fatty acids per polymer chain), suppressing the gel point (*p*_gel_) of the reacting polymer and improving the cross-linking density of the resulting coating.^30^

**Figure 8:**
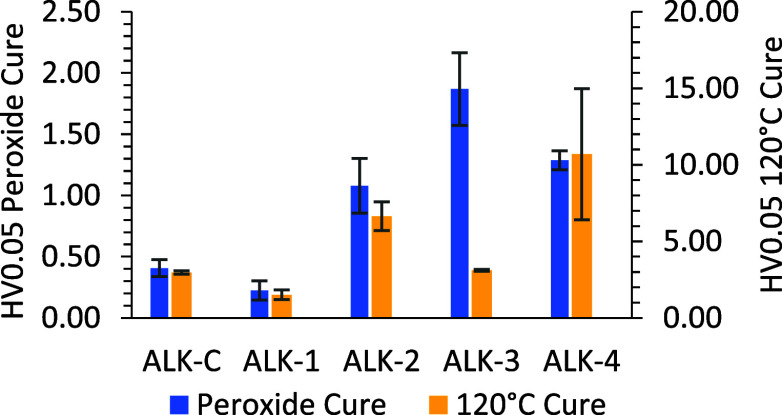
Vickers microhardness results for alkyd coatings cured via peroxide accelerant at ambient temperature and via a 120 °C oven cure.

Increased film-thickness alkyd free-films for physical testing,^31^ were prepared via two methods; (i) curing in an oven at 120 °C for 16 h and (ii) addition of 5 wt % methylethyl ketone peroxide (MEK-P accelerant) to the formulated coating with a 7 day ambient cure. Vickers microhardness testing was used to characterize samples from both cure methods across all 5 alkyd-coating types (ALK-C and ALKs 1-4, Figure 8).

Using both cure methods, the optimized wPET alkyds (ALK-2, -3, and -4) matched or outperformed both the control alkyd coating (ALK-C) and the original wPET-derived coating ALK-1 in microhardness testing. The 120 °C (16 h) cure process delivered overall harder coatings than the “MEK-P accelerated” (ambient) cure in all cases, with the highest hardness values reaching 10.70 for the FDCA/wPET system (Figure 8, ALK-4). These results demonstrate the success of studying and addressing both physical and chemical drying processes in alkyd curing. The cause in difference of microhardness values between peroxide cured compared to oven cured ALK-3 requires further investigation.

**Figure 9:**
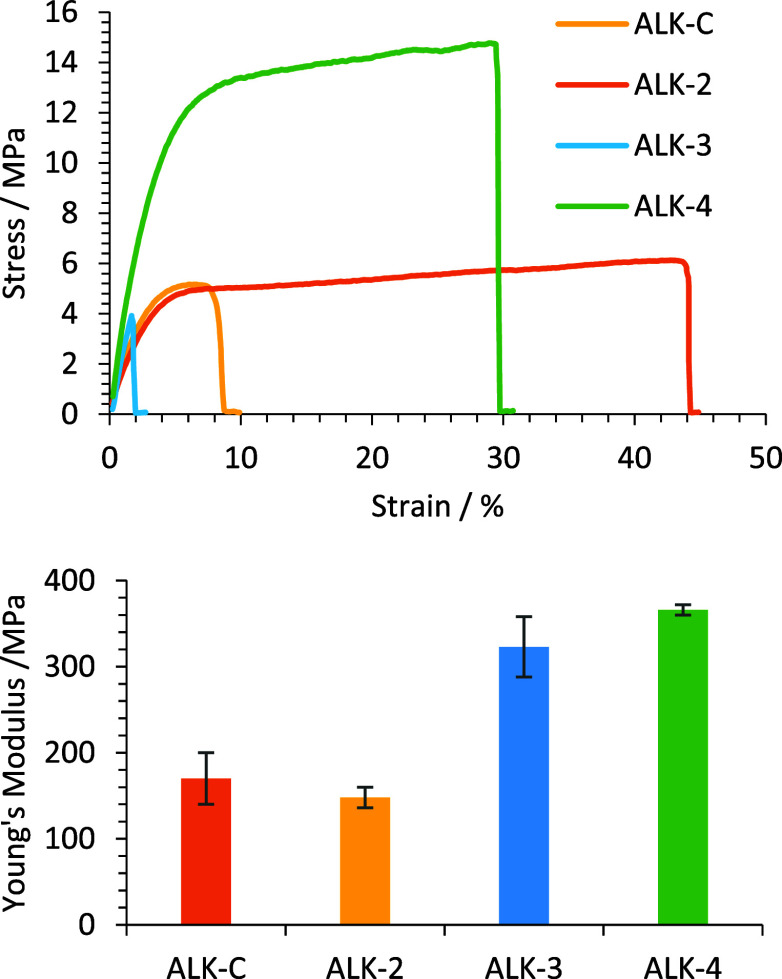
Mechanical test data for oven cured alkyd coatings ALK-C, ALK-2, ALK-3, and ALK-4 including (top) tensile test curves and (bottom) Young’s modulus data.

Oven cured free-film samples (120 °C for 16 h) of ALK-2, -3, and -4 were prepared alongside ALK-C for tensile testing (Figure 9). The rDMT-based ALK-3 exhibited the lowest ultimate tensile strength (UTS) of all samples, supported by a comparatively high Young’s modulus. FDCA-based ALK-4 exhibited the highest UTS and Young’s modulus of the coating samples tested. Notably, ALK-2 and ALK-4 exhibited greater ductility in this test in comparison to ALK-3 and ALK-C, and despite the lower ductility, ALK-3 exhibits comparable UTS to the control ALK-C. Both of the 100% waste/bioderived coatings, ALK-3 (rDMT) and ALK-4 (FDCA) exhibited the highest Young’s modulus values, approximately twice that of the conventional ALK-C. It is noted here that tensile measurements were conducted on oven cured samples; therefore, the lower ductility and UTS of ALK-3 also match the poorer microhardness performance. Observing the ^1^H NMR of UPE-3 (Figure S11 in the Supporting Information), the integral in the region of 3.5–5 ppm is slightly elevated when compared to the region within UPE-2 and UPE-4 (0.54 vs 0.50). Due to the methyl ester protons of rDMT occurring within this region, it is likely that there is some residual methyl ester functionality that was not fully transesterified into the final polymer. This is supported by the polymer UPE-3 having the lowest molecular weight averages of the optimized UPEs, and subsequently, lower functionality may explain the relatively poor performance of ALK-3 in comparison to the other materials tested.

**Figure 10:**
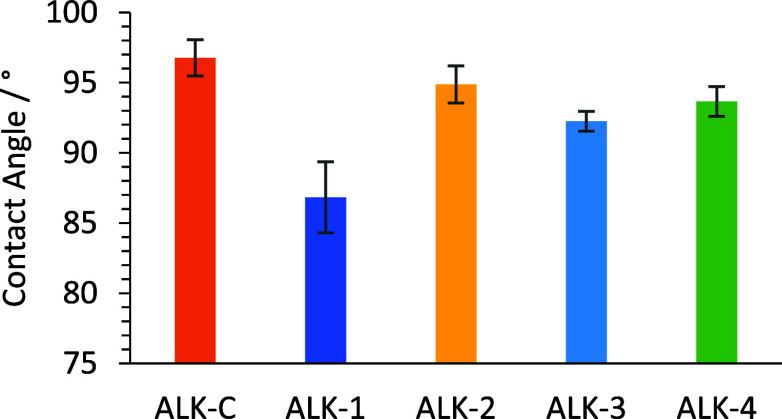
Static water contact angles of oven cured alkyd coating (ALK-C and ALK-1 to -4).

A static water contact angle was used to evaluate the relative hydrophobicity of the cured alkyd coatings by studying the contact line between a 4 μL water droplet and the coating surface. The surface hydrophobicity of the 120 °C (16 h) cured ALK-C and ALK-1 to -4 samples was studied. The slow drying ALK-1 coating exhibited greater hydrophilic character with a static contact angle of 87°, likely attributable to the high hydroxyl excess in the initial polymer (UPE-1) formulation, resulting in increased hydroxyl concentration at the coating surface.^32,33^ The optimized (faster drying and increased hardness) coatings ALK-2, ALK-3, and ALK-4 all showed similar static water contact angles to the control coating ALK-C (Figure 10, entry 1 vs entries 3–4) indicating a similar degree of hydrophobicity to a standard (part petrochemical derived) alkyd coating, which is an important property for the barrier and surface cleaning of industrial alkyd coatings.^34^

**Figure 11:**
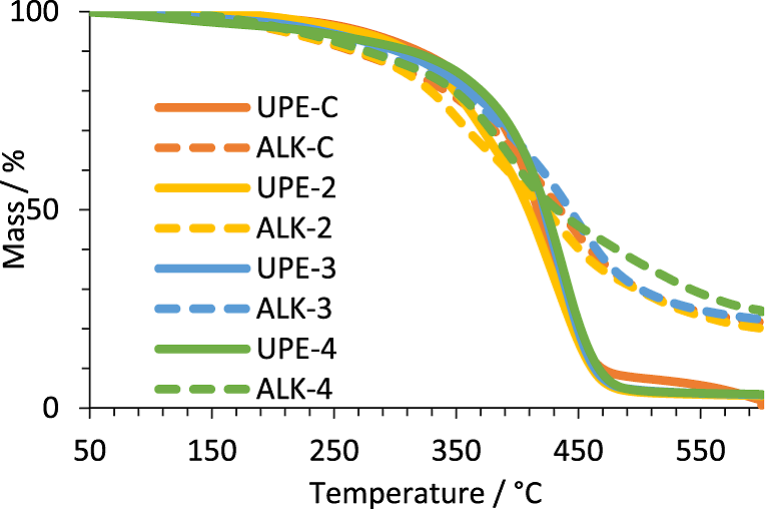
Thermal stability by TGA of polymers and corresponding oven cured (120 °C 16 h) alkyd coatings.

TGA was used to study the thermal stability (Figure 11) of the wPET-derived polymers and corresponding alkyd-coating samples. All experimental samples exhibited similar characteristics to the control UPE-C and ALK-C samples. It was also observed that the thermoset (cross-linked) alkyd-coating samples (ALK) exhibit significantly higher residual mass at 600 °C than the non-cross-linked polymers from which they were derived. This is likely due to (i) the higher thermal stability of thermoset (cross-linked) polymers in comparison to non-cross-linked (thermoplastic) polymers and (ii) a contribution from the noncombustible metallic oxidative drying catalysts used in the alkyd-coating formulations.

## Conclusions

A method is described for the direct conversion of nonrecyclable, contaminated postconsumer wPET into fatty acid-functional polyesters for use in industrial alkyd coatings. The resulting polymers can be 100% derived from waste and biosourced substrates while still delivering physical and material properties comparable to a conventional (part-petrochemical derived) alkyd coating. A convergent methodology (via wPET to rDMT) produces a polyester with desirable hydroxyl excess, drying properties, and physical properties, while the process includes a methodology for >95% efficient uptake of wPET into the resulting polyester. Further to this, the research highlights the benefits of including biobased monomer FDCA in the polyester formulation to improve drying performance, hardness, UTS, and Youngs’ modulus when compared with a petroleum-derived phthalic anhydride-based formulation.

## Supporting Information Available

Spectral data, extended formulation detail for synthesis, application, and testing processes (PDF)
