# Supplementary material for: Conversion of Contaminated Post-Consumer Polyethylene Terephthalate into a Thermoset Alkyd Coating Using Biosourced Monomers
Source: ACS Sustain Chem Eng. 2024 Apr 18;12(17):6485–93. doi: 10.1021/acssuschemeng.3c07560 (PMC11061830; doi:10.1021/acssuschemeng.3c07560)
Supplement: Supplementary file 1 — sc3c07560_si_001.pdf [file sc3c07560_si_001.pdf]

Conversion of Contaminated Post-Consumer PET Into a Thermoset Alkyd Coating Using Biosourced Monomers

Bradley Thomas#, Nicole D. A. Lopez#, James Railton#, Jamal Bousbaa#, Justin J. B. Perry# and Matthew G. Unthank#*

#: Northumbria University, Newcastle upon Tyne NE1 8ST, UK. matthew.unthank@northumbria.ac.uk

*: Corresponding author, Department of Applied Science, Northumbria University, Newcastle upon Tyne NE1 8ST, UK. matthew.unthank@northumbria.ac.uk

Supporting Information

Table S1: Chemicals list. S2
Table S2: Sylfat 2 tall oil fatty acid composition and acid value. S2
Table S3: Table of fatty acid functional ‘unsaturated’ polyester formulations. S3
Table S4: Formulation of UPEs into alkyd coating formulations. S3
Table S5: Titrant and titration solvent details. S4
Table S6: Results of colorimetric acid value determination for UPE-C. S4
Table S7: Reaction components for the synthesis of control polymer UPE-C. S5
Table S8: Reaction components for the synthesis of wPET derived UPE-1. ....S5
Table S9: Reaction components for the synthesis of UPE-2 and determination of theoretical gel point. S6
Table S10: Summary table of tensile and Young's modulus data. S10

Figure S1: UPE-C ATR-FTIR spectra. S7
Figure S2: UPE-1 ATR-FTIR spectra. S7
Figure S3: UPE-2 ATR-FTIR spectra. S8
Figure S4: UPE-3 ATR-FTIR spectra. S8
Figure S5: UPE-4 ATR-FTIR spectra. S9
Figure S6: Tensile test curves of tested alkyd free film samples. S9
Figure S7: Proton NMR spectra of synthesised dimethyl terephthalate. S11
Figure S8: Proton NMR spectra of UPE-C. S12
Figure S9: Proton NMR spectra of UPE-1. ....S13
Figure S10: Proton NMR spectra of UPE-2. ....S14
Figure S11: Proton NMR spectra of UPE-3. ....S15
Figure S12: Proton NMR spectra of UPE-4. ....S16

1. Materials

All materials were purchased from commercial vendors listed in table S1 and used as received without further purification for synthesis unless specifically stated.

Table S1: Chemicals list.

Chemical	CAS No.	Supplier	Purity/Grade
Glycerol	56-81-5	Sigma Aldrich	>98%
Sylfat2 tall oil fatty acids	61790-12-3	Kraton	Technical grade
Methanol	67-56-1	Fisher Scientific	HPLC grade
Toluene	108-88-3	Fisher Scientific	ACS reagent grade
Zinc acetate dihydrate	557-34-6	Alfa Aesar	>98%
Phthalic anhydride	85-44-9	TCI	>98%
2,5-furandicarboxylic acid	3238-40-2	Fluorochem	>96%
Methylethyl ketone peroxide	1338-23-4	Sigma Aldrich	Technical grade
Tetrahydrofuran	109-99-9	Fisher Scientific	GPC grade
Deuterated chloroform (CDCl3)	865-49-6	Cambridge Isotope Laboratories	99.8% + 0.05% w/v TMS
Potassium hydroxide	1310-58-3	Fisher Scientific	SLR pellets
Phenolphthalein	77-09-8	Fisher Scientific	1% in ethanol Indicator grade
Isopropanol	67-63-0	Fisher Scientific	HPLC grade
Methylethylketone oxime	96-29-27	Sigma-Aldrich	99%
White spirit	N/A	Sigma-Aldrich	~17% aromatics basis
Borchers Deca Cobalt 10	N/A	Milliken	N/A
Borchers Octa-Soligen Calcium 10	N/A	Milliken	N/A
Borchers Octa-Soligen Zirconium 18	N/A	Milliken	N/A

Table S2: Sylfat 2 tall oil fatty acid composition and acid value.

Property	Value
Acid value (mg KOH/g)	196
Fatty Acids (%)	96.0
Rosin Acids (%)	1.6
Unsaponifiables (%)	1.0

2. Fatty Acid Functional 'unsaturated' Polyester and Alkyd Coating Formulations

Table S3 contains all monomer formulations of fatty acid functional 'unsaturated' polyesters within this work, in addition to the monomers, all reactions barring UPE-C were also charged with 50 mL of methanol and 0.5 wt% zinc acetate, in accordance with the experimental. It is noted here

that masses of glycerol, tall oil fatty acid and wPET are kept the same, while diacid mass is varied to maintain 0.108 molar equivalents -COOH.

Table S3: Table of unsaturated polyester formulations.

UPE-C Formulation		
Component	Mass (g)	Weight %
Glycerol	11	18.80
Tall oil fatty acids	34	58.12
Phthalic anhydride	13.5	23.08
UPE-1 Formulation		
Component	Mass (g)	Weight %
Glycerol	8.73	14.86
Tall oil fatty acids	30	51.08
wPET	20	34.05
UPE-2 Formulation		
Component	Mass (g)	Weight %
Glycerol	8.73	13.08
Tall oil fatty acids	30	44.96
wPET	20	29.97
Phthalic anhydride	8	11.99
UPE-3 Formulation		
Component	Mass (g)	Weight %
Glycerol	8.73	12.70
Tall oil fatty acids	30	43.65
wPET	20	29.10
Dimethyl terephthalate	10	14.55
UPE-4 Formulation		
Component	Mass (g)	Weight %
Glycerol	8.73	13.08
Tall oil fatty acids	30	44.96
wPET	20	29.97
2,5-furandicarboxylic acid	8	11.99

Table S4: Formulation of UPEs into alkyd coating formulations.

**Alkyd Coating Formulation Component**	**Parts**	**%**
UPE (solvent free)	10	50.71
10wt% solution of cobalt auto-oxidation catalyst	0.25	1.27
10wt% solution of calcium auto-oxidation catalyst	1	5.07
18wt% solution of zirconium auto-oxidation catalyst	1.4	7.10
Methylethyl ketone oxime	0.4	2.03
White Spirit	6.67	33.82
Total	19.72	100.00

3. Determination of Acid Number of UPE-C

Acid value was determined in accordance with ASTM D974: "Standard Test Method for Acid and Base Number by Color-Indicator Titration"

Table S5: Titrant and titration solvent details.

Titrant	
Mass KOH pellets (g)	0.2862
Isopropanol (mL)	250
mg KOH/mL	1.145

Titration solvent	
Toluene (mL)	250
Isopropanol (mL)	247.5
Deionised water (mL)	2.5

Table S6: Results of colorimetric acid value determination for UPE-C.

Run	Blank titre (mL)	Sample mass (g)	Titre (mL)	Titre - blank (mL)	Acid value (mg KOH/g)
1	2.3	1.0284	16.8	14.5	16.60
2	2.6	1.0653	17.4	14.8	16.94
3	2.4	0.9520	16.5	14.1	16.14

Average Acid value: (mg KOH/g)	16.56
Standard deviation:	0.328

4. Theoretical Calculations for Degree of Polymerization of UPE-C & UPE-1 and Gel Point of UPE-2

Table S7: Reaction components for the synthesis of control polymer UPE-C.

Reagent	Mass (g)	Moles	Functionality	Equivalents
Glycerol	11	0.119	3	0.358
TOFA	34	0.121	1	0.121
Phthalic anhydride	13.5	0.091	2	0.182

$$Total - OH\ equivalents\ (e_b) = 0.358$$

$$Total - COOH\ equivalents\ (e_a) = (0.121 + 0.182) = 0.303$$

$$Average\ functionality\ f_{av} = \frac{2e_a}{m_0}\ where:$$

$$m_0 = total\ moles\ of\ reactants$$

$$\therefore\ f_{av} = \frac{2 \times 0.303}{(0.119 + 0.121 + 0.091)} = 1.831$$

$$Water\ loss\ adjusted\ polymer\ mass\ W_p = \frac{W_0 - 18e_a + 18e_{an}}{1 - 18\left(\frac{AN_p}{56100}\right)}\ where:$$

$$W_0 = initial\ reaction\ mass$$

$$e_{an} = anhydride\ equivalents$$

$$AN_p = Determined\ residual\ acid\ value$$

$$\therefore\ W_p = \frac{58.5 - (18 \times 0.303) + (18 \times 0.091)}{1 - 18(\frac{16.6}{56100})} = 54.977$$

$$Extent\ of\ reaction\ p_a = \frac{e_a - (\frac{AN_p \times W_p}{56100)})}{e_a}$$

$$\therefore p_a = \frac{0.303 - (\frac{16.6 * 54.977}{56100})}{0.303} = 0.9463$$

$$Theoretical\ Degree\ of\ polymerization\ Xn = \frac{2}{2 - pf_{av}}$$

$$\therefore Xn\ for\ UPE - C = \frac{2}{2 - 0.9463 * 1.831} = 7.482$$

Table 8: Reaction components for the synthesis of wPET derived UPE-1.

Reagent	Mass (g)	Moles	Functionalit y	Equivalent s
Glycerol	11	0.119	3	0.358
TOFA	34	0.121	1	0.121
Ethylene glycol	5.41	0.087	2	0.174
Terephthalic acid	14.49	0.087	2	0.174

$$Total - OH\ equivalents\ (e_b) = 0.458$$

$$Total - COOH\ equivalents\ (e_a) = (0.107 + 0.174) = 0.281$$

$$Average\ functionality\ f_{av} = \frac{2e_a}{m_0}\ where:$$

$$m_0 = total\ moles\ of\ reactants$$

$$\therefore f_{av} = \frac{2 \times 0.281}{(0.095 + 0.107 + 0.087 + 0.087)} = 1.495$$

$$Extent\ of\ reaction\ p = \frac{2}{f}\left(1 - \frac{1}{Xn}\right)$$

$$p = \frac{2}{1.495}\left(1 - \frac{1}{Xn}\right)$$

$$Assuming\ p = 1\ \therefore 1 = \frac{2}{1.495}\left(1 - \frac{1}{Xn}\right)$$

$$Theoretical\ maximum\ degree\ of\ polymerization\ Xn\ for\ UPE - 1 = \frac{2}{2 - 1.495} = 3.960$$

Table S9: Reaction components for the synthesis of UPE-2 and determination of theoretical gel point.

UPE-2		Group	Mw	Functionality	Mass (g)	Moles	Moles -OH	Moles -COOH
B3	Glycerol	OH	92.09	3	8.73	0.095	0.284	
A1	TOFA	COOH	281	1	30	0.107		0.107
B2	Ethylene glycol	OH	62.07	2	5.41	0.087	0.174	
A2	Terephthalic acid	COOH	166.13	2	14.49	0.087		0.174
A2	Phthalic anhydride	COOH	148.1	2	8	0.054		0.108
				Sum	66.63		0.459	0.389

$$p_c = \frac{1}{\{r(f_{w,A} - 1)(f_{w,B} - 1)\}^{1/2}}$$

$$where:$$

$$p_c = theoretical\ gel\ point$$

$$r = ratio\ between\ total\ A\ and\ B\ groups$$

$$f_{w,A} = weight\ average\ functionality - COOH$$

$$f_{w,B} = weight\ average\ functionality - OH$$

$$f_{w,A} = \frac{\sum f_{A_i}^2 N_{A_i}}{\sum f_{A_i} N_{A_i}}$$

$$f_{w,B} = \frac{\sum f_{B_j}^2 N_{B_j}}{\sum f_{B_j} N_{B_j}}$$

$$r = \frac{1 \times 0.107 + 2 \times 0.087 + 2 \times 0.054}{3 \times 0.095 + 2 \times 0.087} = 0.849$$

$$f_{w,A} = \frac{1^2 \times 0.107 + 2^2 \times 0.087 + \ 2^2 \times 0.054}{1 \times 0.107 + 2 \times 0.087 + 2 \times 0.054} = 1.726$$

$$f_{w,B} = \frac{3^2 \times 0.095 + 2^2 \times 0.087}{3 \times 0.095 + 2 \times 0.087} = 2.619$$

$$Theoretical\ gel\ point\ p_c = \frac{1}{\{0.849(1.726 - 1)(2.619 - 1)\}^{1/2}} = 1.001$$

5. Attenuated Total Reflectance Fourier Transform Infrared Spectra of Unsaturated Polyesters

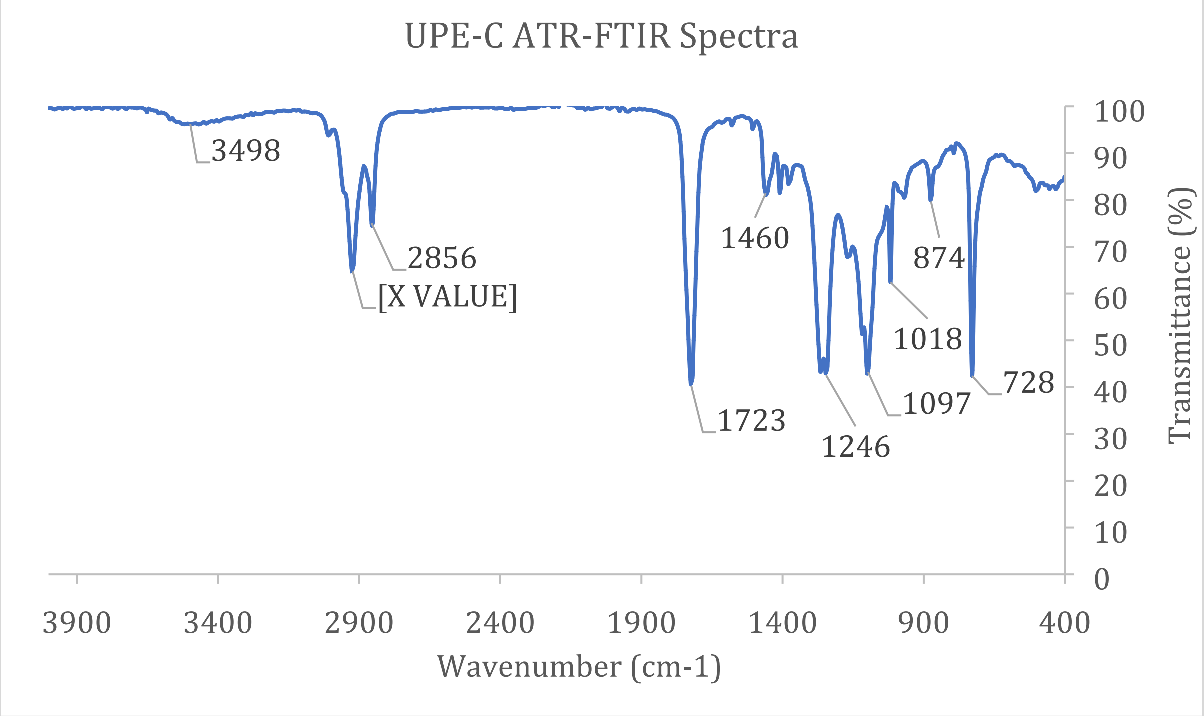

Figure S1: UPE-C ATR-FTIR spectra.

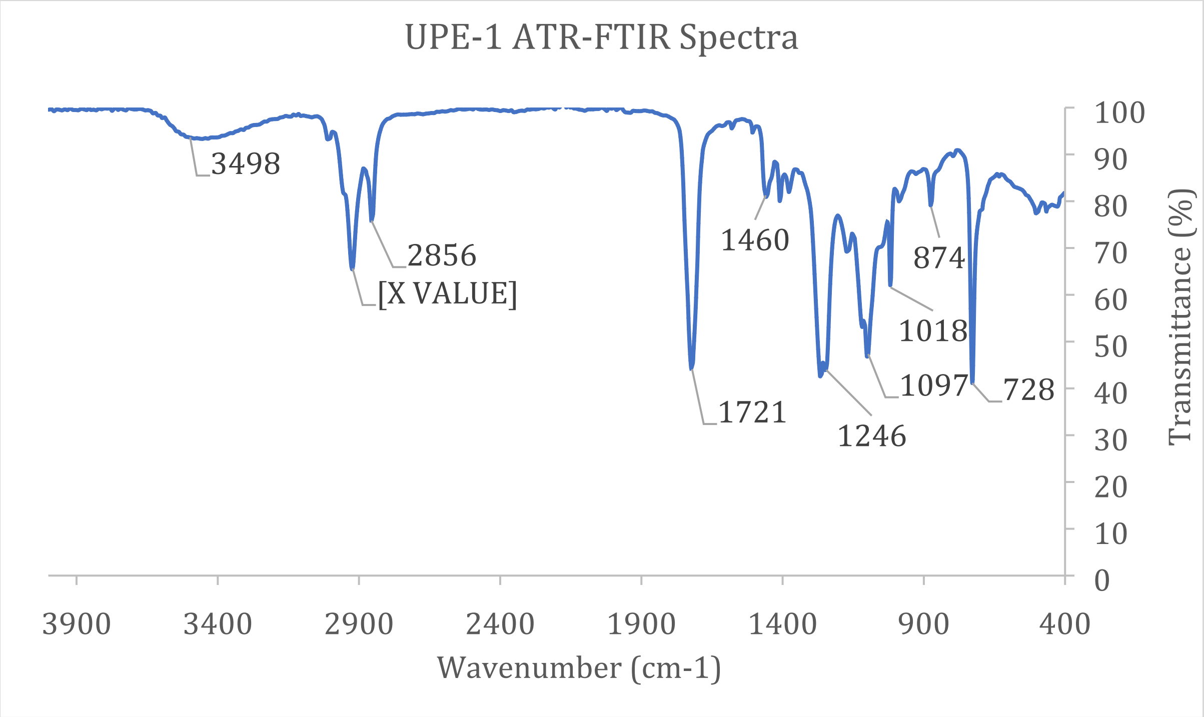

Figure S2: UPE-1 ATR-FTIR spectra.

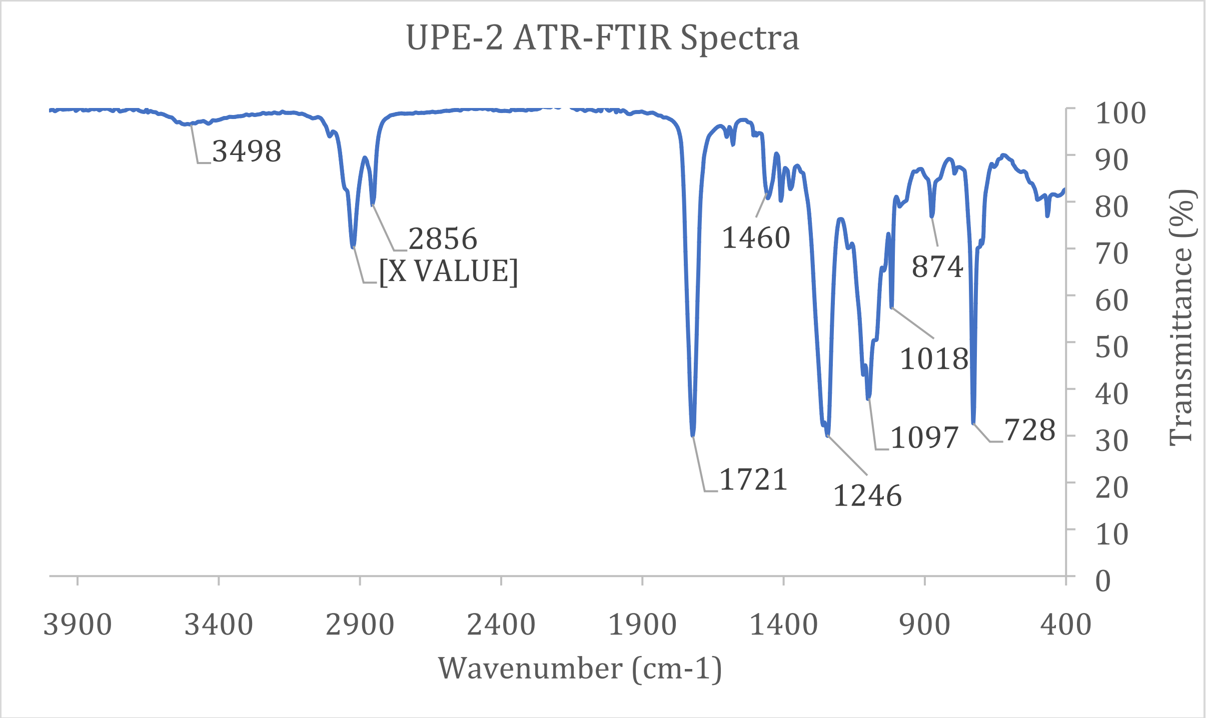

Figure S3: UPE-2 ATR-FTIR spectra.

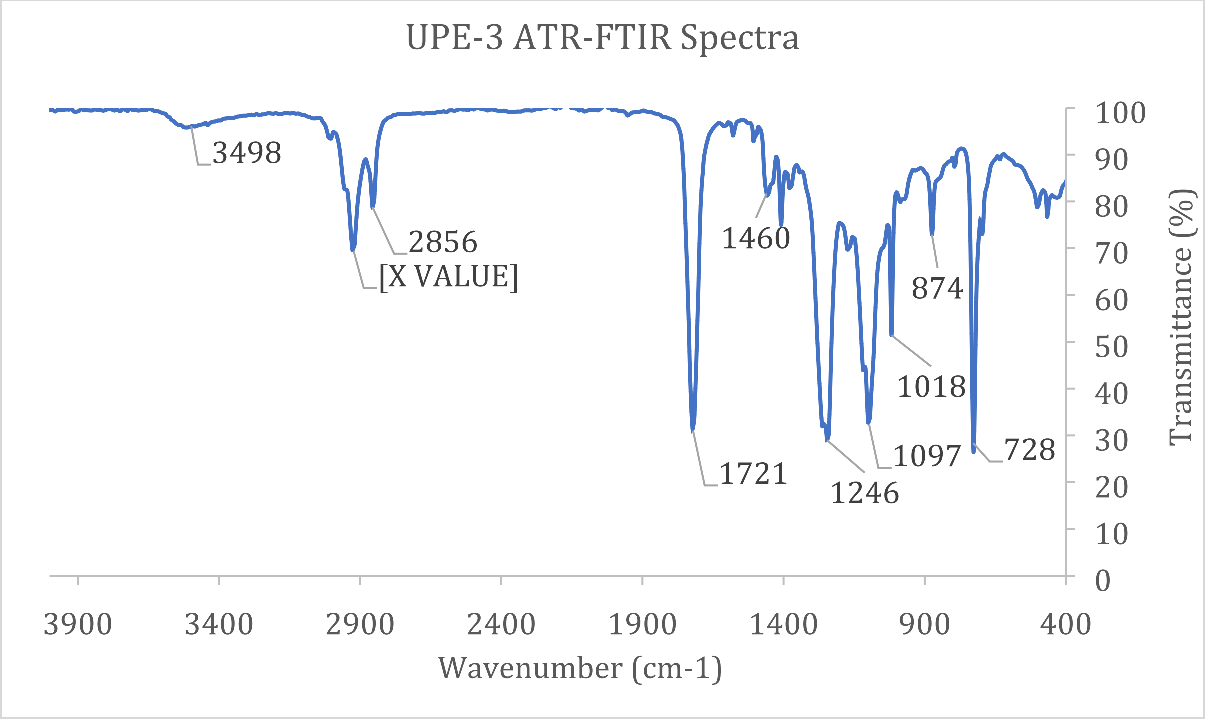

Figure S4: UPE-3 ATR-FTIR spectra.

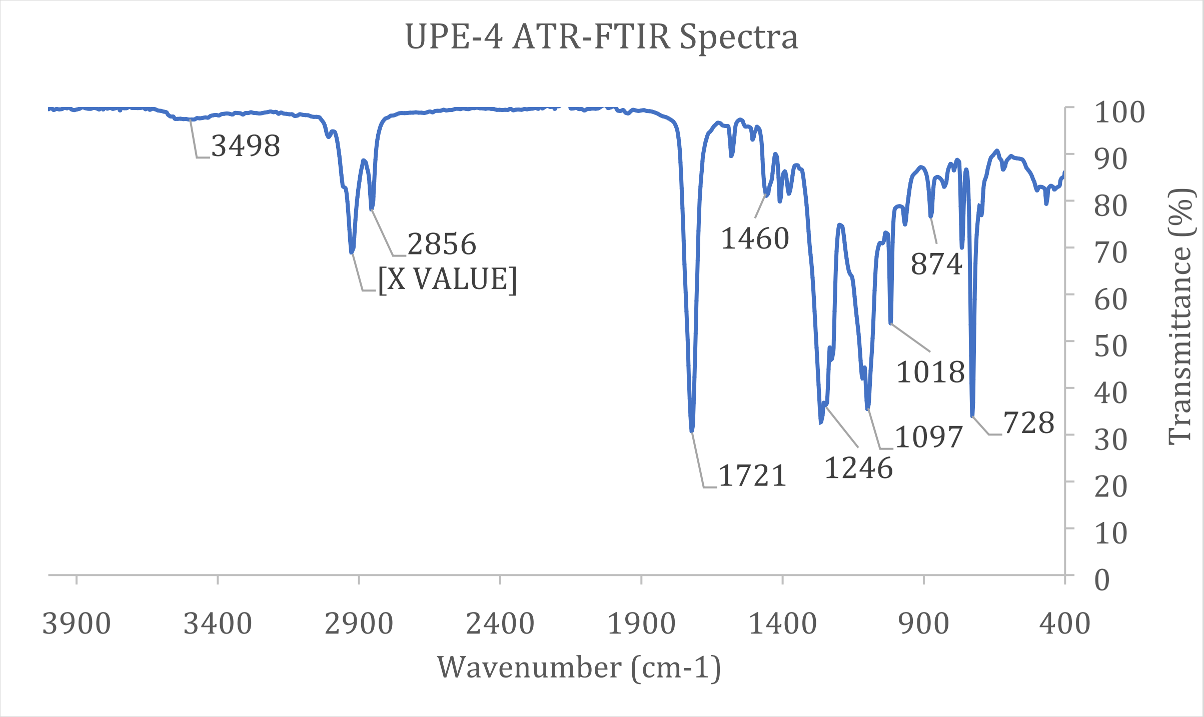

Figure S5: UPE-4 ATR-FTIR spectra.

6. Tensile Testing Data

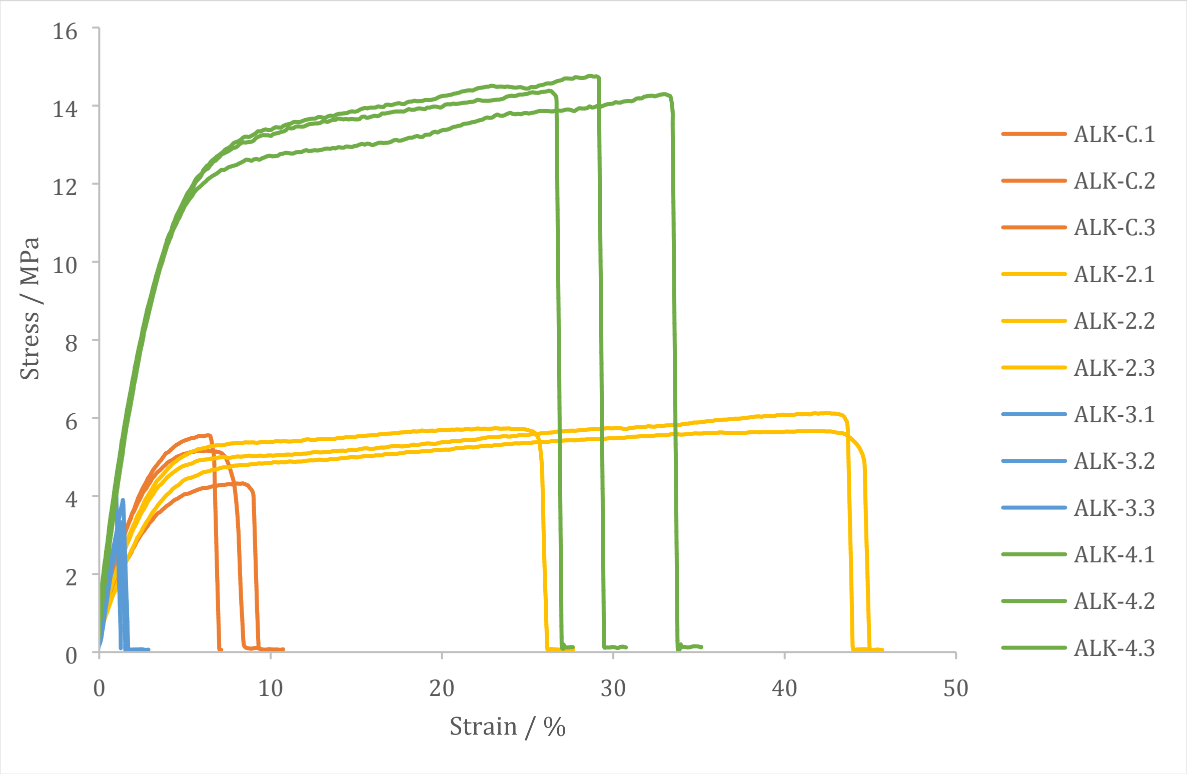

Figure S6: Tensile test curves of tested alkyd free film samples.

Table S10: Summary table of tensile and Young's modulus data.

UPE-C					
Run	Ultimate Tensile Strength (N)	Elongation at Break (mm)	Ultimate Tensile Stress (MPa)	Strain at break (%)	Youngs Modulus (MPa)
1	4.29	2.78	4.32	9.27	127.3
2	5.52	2.04	5.56	6.80	199.7
3	5.13	2.30	5.17	7.67	184.0
Average	4.98	2.37	5.02	7.91	170.3
Standard Deviation	0.51	0.31	0.52	1.02	31.10
UPE-2					
Run	Ultimate Tensile Strength (N)	Elongation at Break (mm)	Ultimate Tensile Stress (MPa)	Strain at break (%)	Youngs Modulus (MPa)
1	7.27	13.12	11.06	43.73	145.6
2	6.73	13.16	11.09	43.87	133.9
3	6.81	7.70	6.49	25.67	164.0
Average	6.94	11.33	9.55	37.76	147.8
Standard Deviation	0.24	2.56	2.16	8.55	12.40
UPE-3					
Run	Ultimate Tensile Strength (N)	Elongation at Break (mm)	Ultimate Tensile Stress (MPa)	Strain at break (%)	Youngs Modulus (MPa)
1	3.83	0.38	0.39	1.25	372.2
2	3.18	0.46	0.48	1.52	306.9
3	3.74	0.50	0.53	1.68	290.4
Average	3.58	0.45	0.47	1.48	323.2
Standard Deviation	0.29	0.05	0.06	0.18	35.31
UPE-4					
Run	Ultimate Tensile Strength (N)	Elongation at Break (mm)	Ultimate Tensile Stress (MPa)	Strain at break (%)	Youngs Modulus (MPa)
1	8.39	8.09	13.88	26.97	368.0
2	8.61	8.83	15.15	29.43	372.0
3	8.34	10.12	17.36	33.73	358.0
Average	8.45	9.01	15.46	30.04	366.0
Standard Deviation	0.12	0.84	1.44	2.80	5.89

7. Proton Nuclear Magnetic Resonance Spectra

6.1: Recycled dimethyl terephthalate

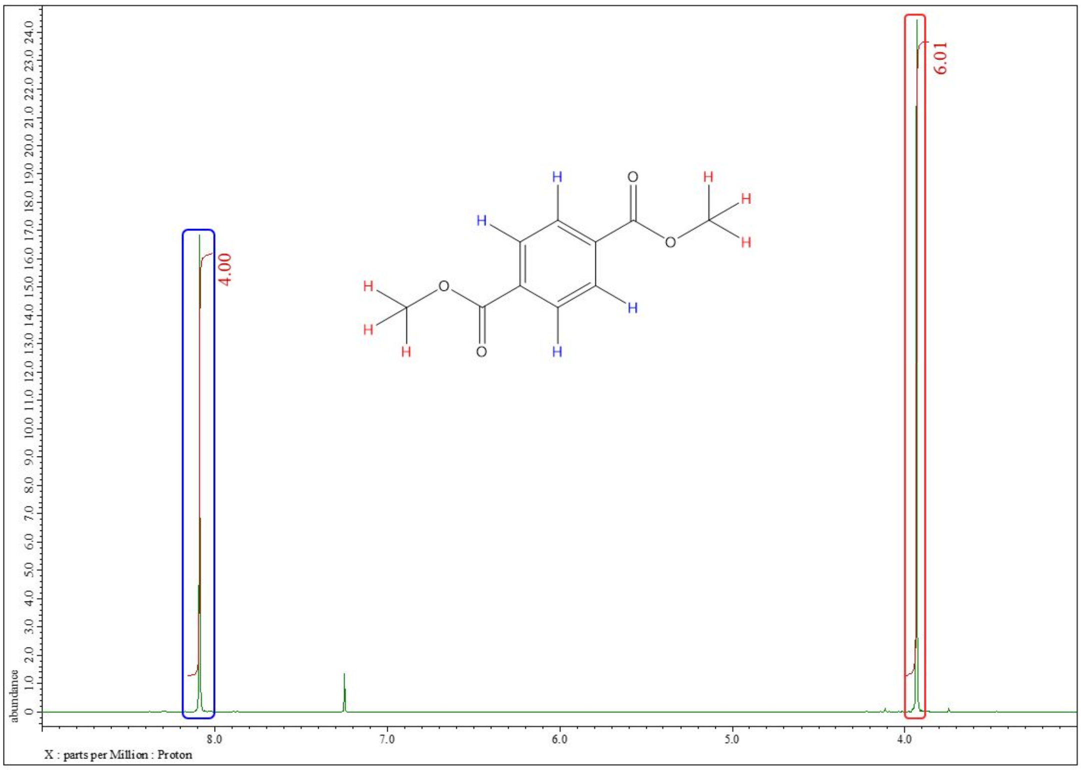

Figure S7: Proton NMR spectra of synthesised dimethyl terephthalate.

6.2: Fatty acid functional ‘unsaturated’ Polyester Resins

For the assignment of proton environments in the following NMR spectra, linoleic acid has been used as a general fatty acid, as Sylfat 2 used in the polymerizations also contains other unsaturated acids, as well as rosin acids.

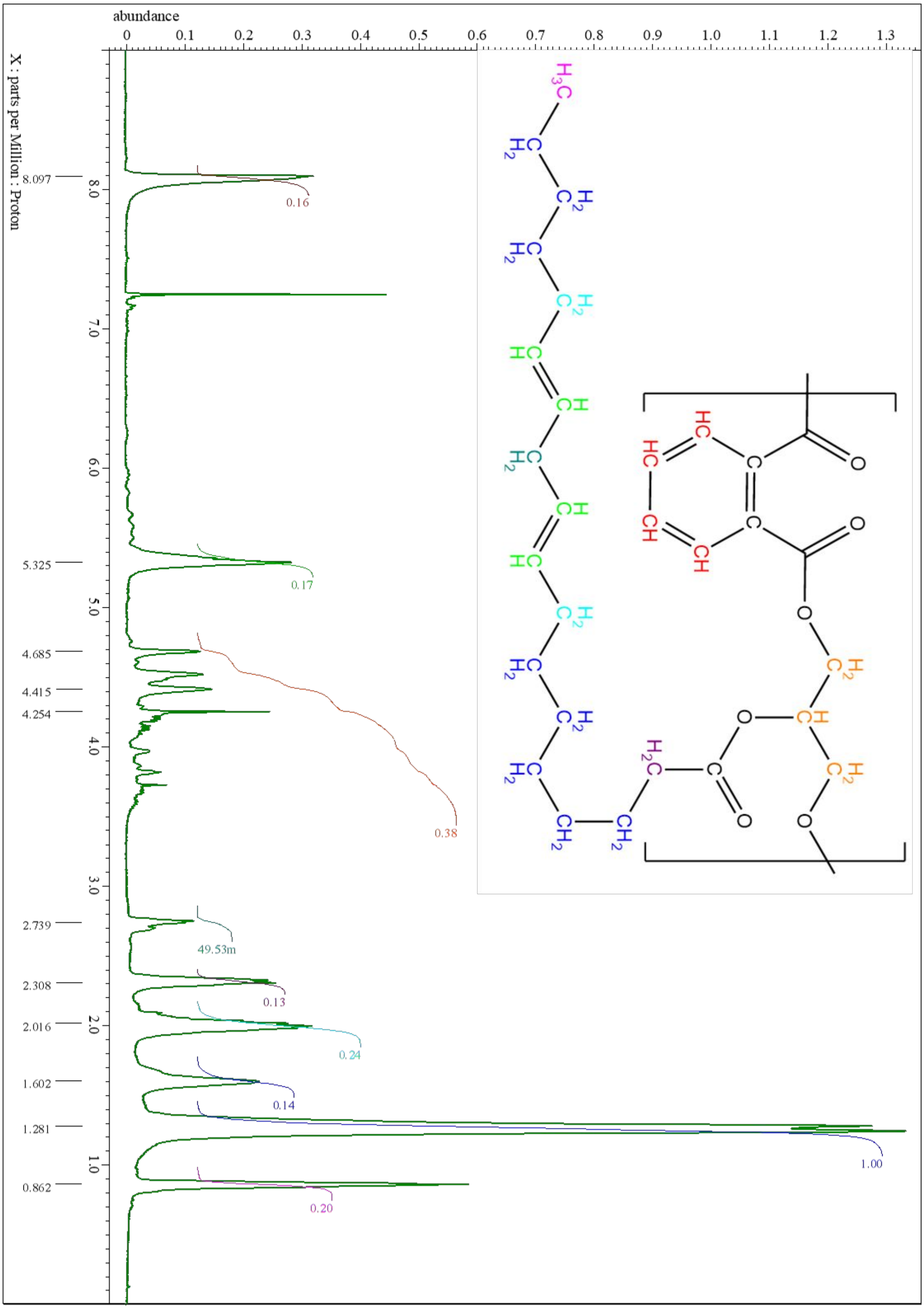

Figure S8: Proton NMR spectra of UPE-C.

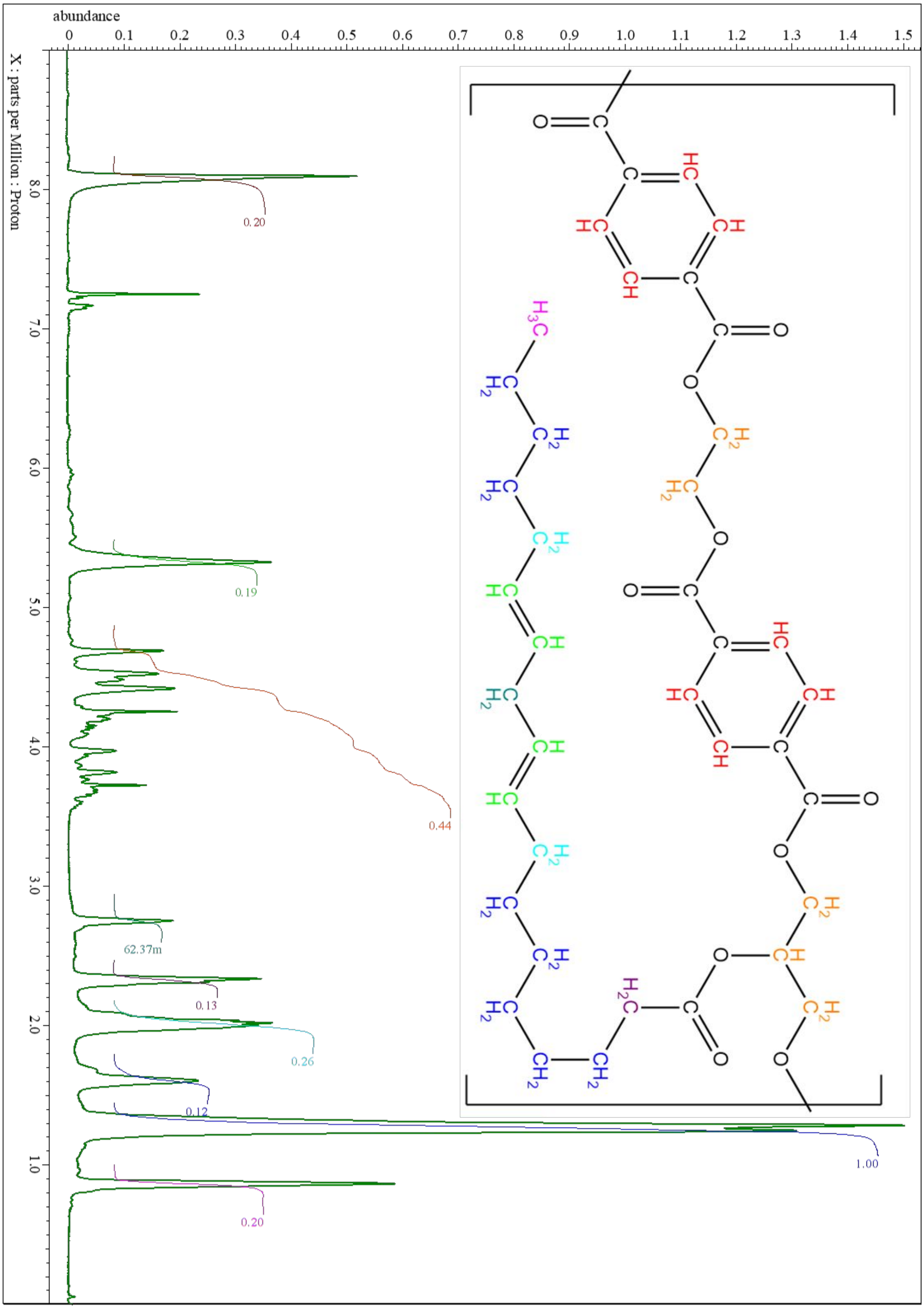

Figure S9: Proton NMR spectra of UPE-1, the peak at 8 ppm indicates the presence of aromatic protons. These would not be present were there no reaction between PET and the other components, especially due to PETs very poor solubility in deuterated chloroform.

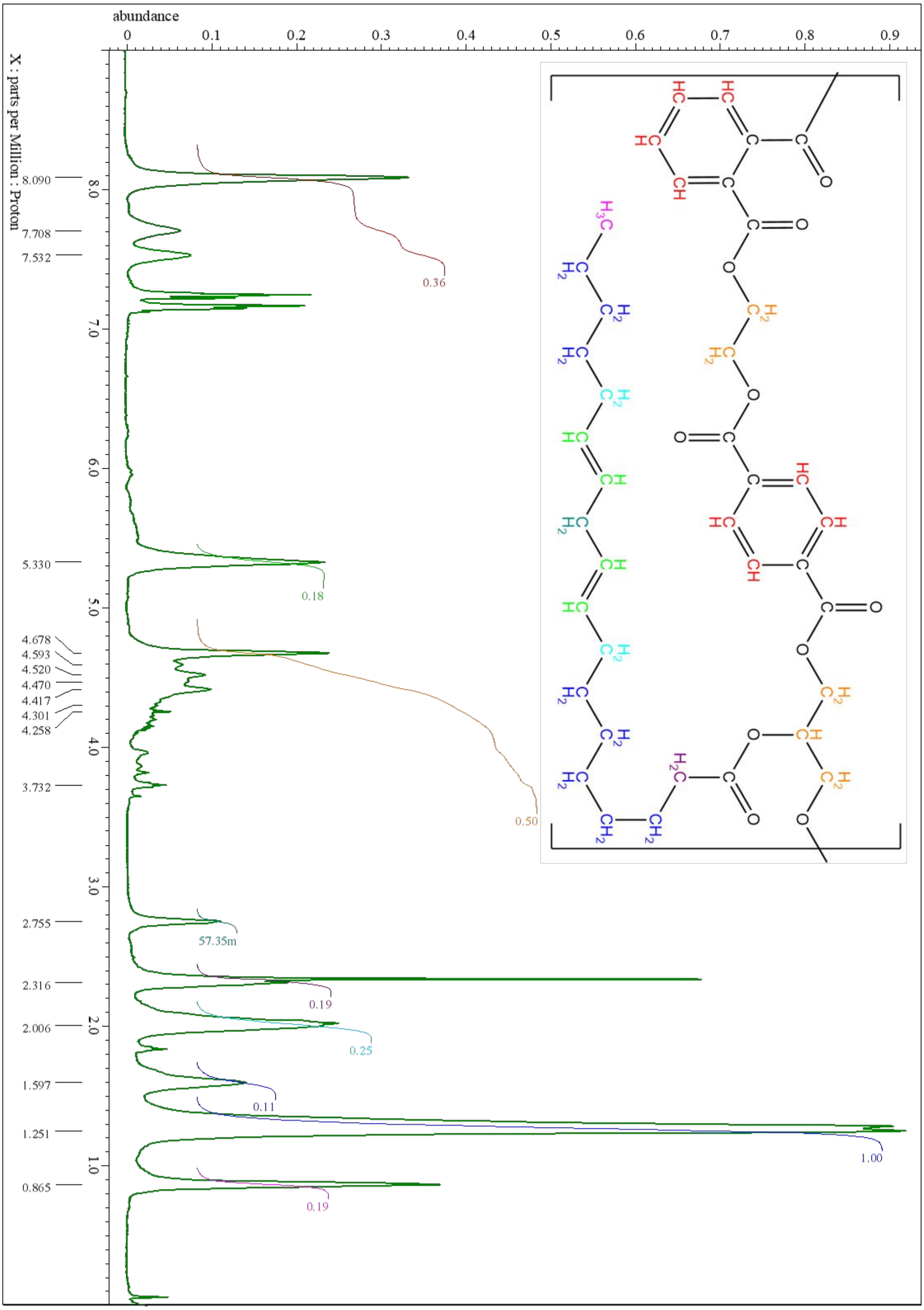

Figure S10: Proton NMR spectra of UPE-2.

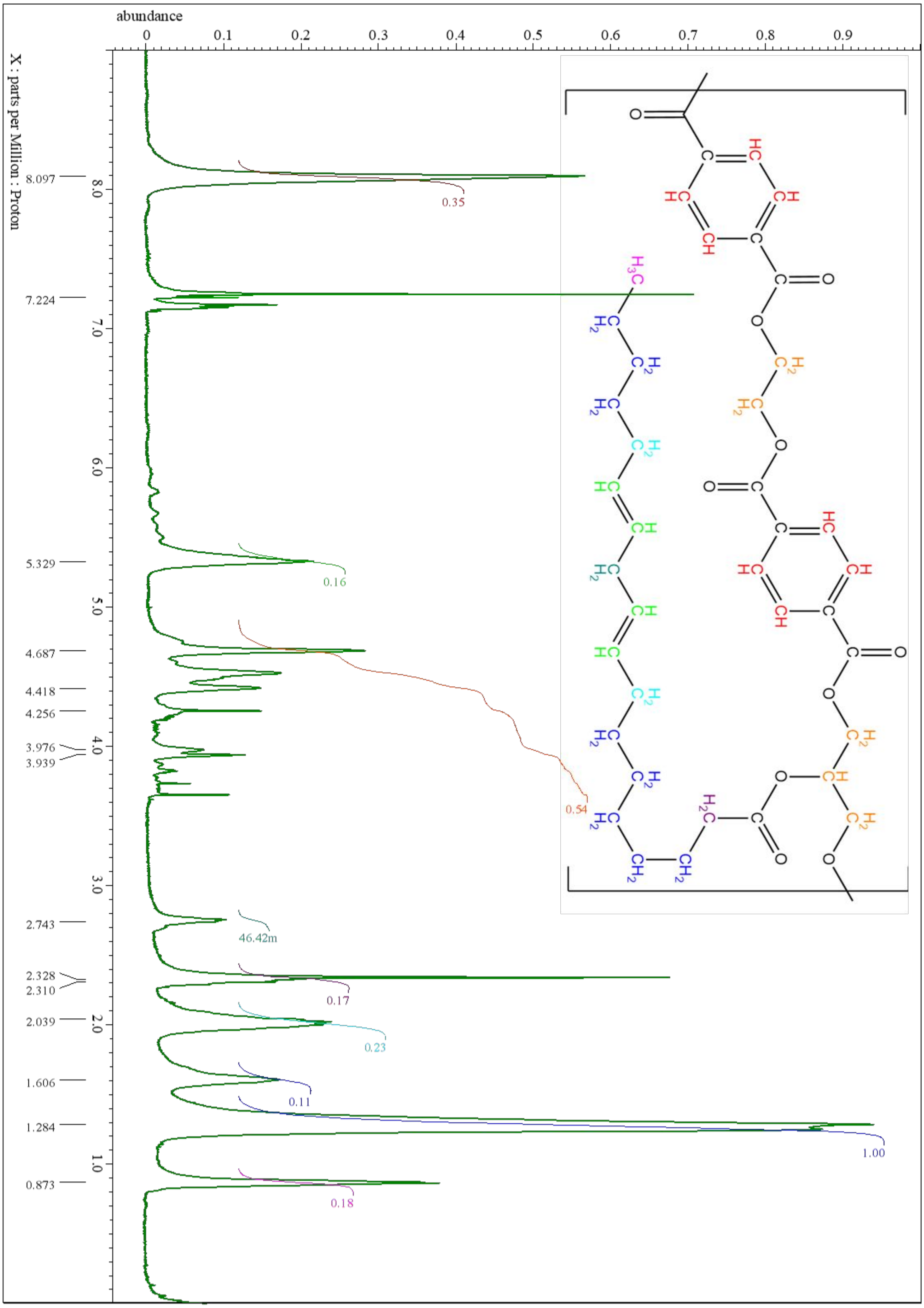

Figure S11: Proton NMR spectra of UPE-3.

Note same repeat unit is given as in UPE-1 for simplicity, but integral of aromatic peaks at 8ppm is 0.35 vs UPE-1 at 0.2, highlighting the inclusion of dimethyl terephthalate.

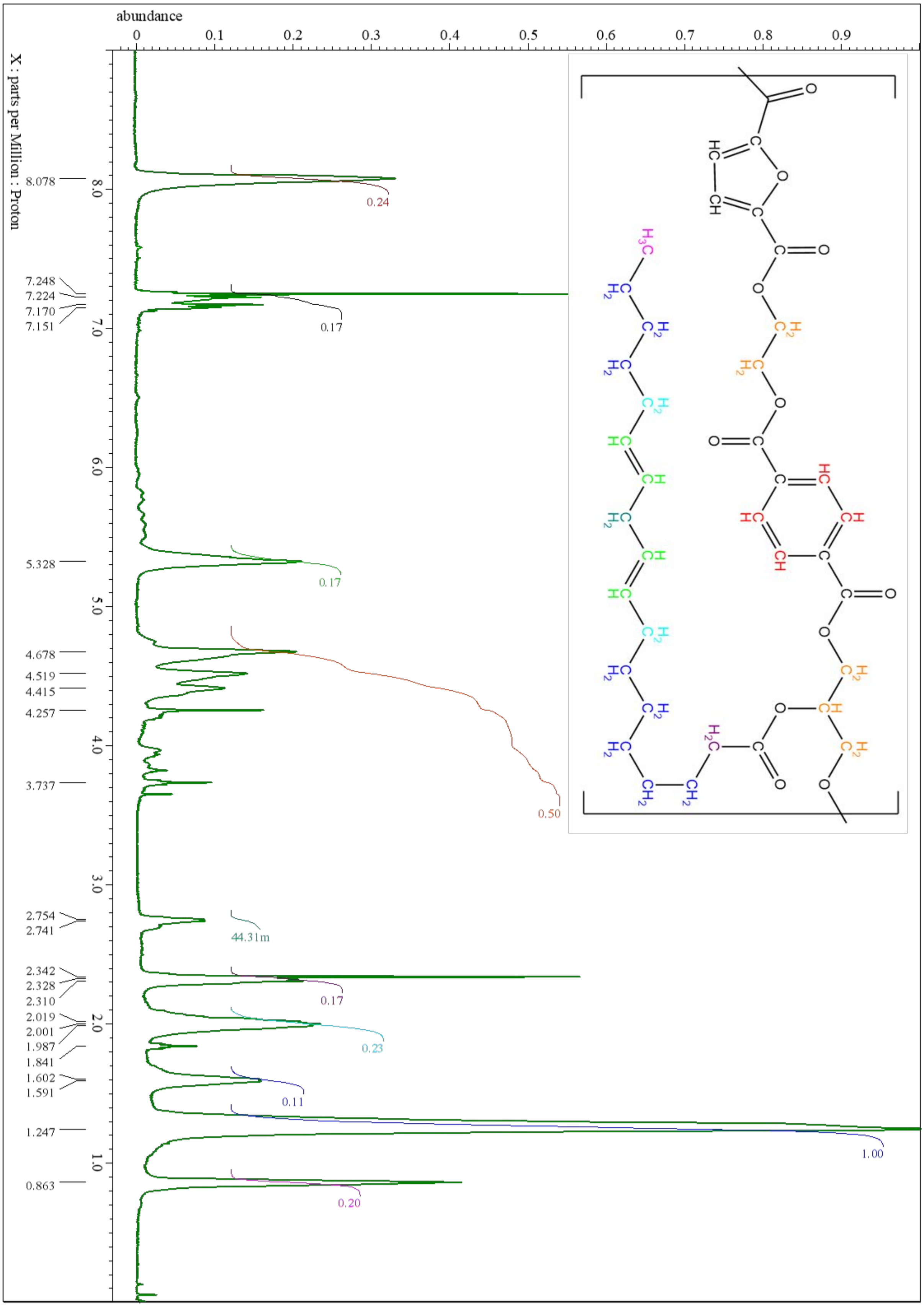

Figure S12: Proton NMR spectra of UPE-4.

Note aromatic furan peaks occurring at ~7.1ppm are masked by $CHCl_3$ peak and potential residual toluene.
